# Antitumor Effect of Simvastatin in Combination With DNA Methyltransferase Inhibitor on Gastric Cancer *via* GSDME-Mediated Pyroptosis

**DOI:** 10.3389/fphar.2022.860546

**Published:** 2022-04-20

**Authors:** Ying Xia, Yong Jin, Daxiang Cui, Xia Wu, Cunfeng Song, Weilin Jin, Hai Huang

**Affiliations:** ^1^ Center for Clinical Laboratories, The Affiliated Hospital of Guizhou Medical University, Guiyang, China; ^2^ Department of Pathophysiology, School of Basic Medical Science, Guizhou Medical University, Guiyang, China; ^3^ Department of Clinical Laboratory, The First Affiliated Hospital of Guizhou University of Traditional Chinese Medicine, Guiyang, China; ^4^ School of Clinical Laboratory Science, Guizhou Medical University, Guiyang, China; ^5^ Shanghai Engineering Research Center for Intelligent Diagnosis and Treatment Instrument, Department of Instrument Science and Engineering, School of Electronic Information and Electrical Engineering, Institute of Nano Biomedicine and Engineering, Shanghai Jiao Tong University, Shanghai, China; ^6^ Guizhou Provincial People’s Hospital, Guiyang, China; ^7^ Institute of Cancer Neuroscience, Medical Frontier Innovation Research Center, The First Hospital of Lanzhou University, The First Clinical Medical College of Lanzhou University, Lanzhou, China

**Keywords:** GSDME, pyroptosis, DNA methyltransferase inhibitor, gastric cancer, simvastatin

## Abstract

Gasdermin E (GSDME) is one of the executors of pyroptosis, a type of programmed lytic cell death, which can be triggered by caspase-3 activation upon stimulation. Silenced GSDME expression due to promoter hypermethylation is associated with gastric cancer (GC), which is confirmed in the present study by bioinformatics analysis and methylation-specific PCR (MSP) test of GC cell lines and clinical samples. GC cell lines and mouse xenograft models were used to investigate the pyroptosis-inducing effect of the common cholesterol-depleting, drug simvastatin (SIM), allied with upregulating GSDME expression by doxycycline (DOX)- inducible Tet-on system or DNA methyltransferase inhibitor 5-Aza-2′-deoxycytidine (5-Aza-CdR). Cell viability assessment and xenograft tumour growth demonstrated that the tumour inhibition effects of SIM can be enhanced by elevated GSDME expression. Morphological examinations and assays measuring lactate dehydrogenase (LDH) release and caspase-3/GSDME protein cleavage underlined the stimulation of pyroptosis as an important mechanism. Using short hairpin RNA (shRNA) knockdown of caspase-3 or GSDME, and caspase-3-specific inhibitors, we provided evidence of the requirement of caspase-3/GSDME in the pyroptosis process triggered by SIM. We conclude that reactivating GSDME expression and thereby inducing cancer cell-specific pyroptosis could be a potential therapeutic strategy against GC.

## Introduction

Gastric cancer (GC) is ranked third in cancer mortality and poses a serious threat to human health (Mattiuzzi and Lippi, 2020). To date, surgery and chemoradiotherapy remain the first-line treatment towards GC, but many patients still suffer from poor prognosis and low 5-year survival rate due to severe adverse effects and acquired drug resistance (Yang et al., 2022). Induction of pyroptosis has been emerging as a novel therapeutic strategy against a variety of malignant tumours including GC (Zhou and Fang, 2019; Tan et al., 2021a; Li et al., 2021; Shao et al., 2021), because of its dual function of cytotoxicity and immunogenicity.

Pyroptosis is a gasdermin (GSDM)-dependent programmed cell death featuring membrane rupture formed by translocated GSDM fragments originated from caspase cleavage (Xia et al., 2020). One important characteristic of pyroptosis is the release of cellular contents such as pro-inflammatory cytokines interleukin-1β (IL-1β) and interleukin-18 (IL-18), which actively regulate the immune profile of the tumour microenvironment (Tan et al., 2021b). Gasdermin D (GSDMD) and gasdermin E (GSDME) are the best-studied pyroptotic effectors in the GSDM family. Their sheared N-terminals perforate cell membrane in a similar manner but differ in the activation cascades (Jorgensen and Miao, 2015; Hou et al., 2021). The anti-tumour action of Cucurbitacin B was found to involve the activation of GSDMD-dependent pyroptosis of non-small cell lung cancer (NSCLC) cells *in vitro* and *in vivo* (Yuan et al., 2021). GSDME, located on chromosome 7p15 and also named deafness autosomal dominant 5 (DFNA5) due to its association with hereditary hearing loss (Van Laer et al., 1998), has been implicated as a putative tumour suppressor (Rogers et al., 2017; Wang et al., 2017). Cleaved by the activated caspase-3, the N-terminal of GSDME (GSDME-N) inserts into the membrane lipids and forms pores to trigger pyroptosis (Wang et al., 2017).

In contrast to the substantial investment of time and resources into developing a new drug from scratch repurposing approved drugs can significantly accelerate the clinical translation of basic research (Pushpakom et al., 2019), as exemplified in the quest for drugs to induce pyroptosis. The histamine 2 antagonist famotidine is found to induce GSDME-, not GSDMD-, mediated cell pyroptosis by activation of NLRP3 inflammasome form, leading to increase Caspase-1 activation and IL-18 release in GC cells (Huang et al., 2021). In recent years, studies and clinical trials exploiting the preventive and therapeutic potential of statin in cancer treatment are flourishing. Statin is a class of HMG-CoA reductase inhibitors and the most commonly prescribed cholesterol-reducing drugs thanks to their safety, efficacy, and low cost (Jiang et al., 2021a). In particular, it was shown that simvastatin (SIM), one of the six statin medicines, elicits its anti-cancer effects through not only lowering of cholesterol content, but also activation of caspase-3 (Deng et al., 2019; Alhakamy et al., 2020; Sun et al., 2020) and consequently apoptosis or pyroptosis (Peng et al., 2020; Xu et al., 2021). Moreover, the SIM-induced pyroptosis is confirmed to be cancer cell-specific in lung cancer, without causing toxicity to normal cells (Wang et al., 2018).

However, the death of tumour cells *via* pyroptosis is often dampened by epigenetic silencing of GSDME, as hypermethylation of GSDME promoter is found in about 52%–65% of primary cancers (Op de Beeck et al., 2011) including colorectal cancer (Kim et al., 2008a; Yokomizo et al., 2012) and GC (Akino et al., 2007). Studies have used hypomethylating agents such as decitabine (DAC) (Fan et al., 2019) or 5-aza-2′-deoxycytidine (5-Aza-CdR) (Kim et al., 2008b) to restore the transcription of the GSDME gene and reinstate the sensitivity of various cancer cells, including GC cells, to chemotherapy (Akino et al., 2007). In this study, using GC cell lines MGC-803 and HGC-27 and mouse xenograft GC models, we aim to seek the optimal antitumor effects of SIM- induced pyroptosis, in tandem with upregulating GSDME gene expression.

## Materials and Methods

### Patient Samples

The surgical specimens of 20 paired GC tissues and adjacent non-cancerous tissues were collected from the Departments of Gastrointestinal Surgery, Affiliated Hospital of Guizhou Medical University. Use of the clinical samples were approved by the Affiliated Hospital of Guizhou Medical University Ethics Committee, approval number: 2020 (106). Written informed consent was acquired from all patients. The clinicopathological characteristics of the patients are shown in Supplementary Table S1.

### Cell Culture and Reagents

The GC cell lines AGS, MKN45, and HGC-27 were obtained from the Chinese Academy of Sciences (Shanghai, China). The GC cell line MGC-803 was from the Institute of Nano Biomedicine and Engineering, Shanghai Engineering Research Centre (Shanghai, China). The cells were cultured in Dulbecco’s modified Eagle’s medium (HyClone, Logan, UT, United States) containing 10% foetal bovine serum (FBS, Gibco BRL, Gaithersburg, MD, United States), 100 U/ml penicillin, and 100 mg/ml streptomycin (Invitrogen, Carlsbad, CA, United States). All cells were incubated at 37°C in 5% CO_2_. SIM (Cat. HY-17502) and 5-Aza-CdR (Cat. HY-0004) were purchased from MedChemExpress (Shanghai, China). Doxorubicin hydrochloride (DOX, Cat. ST039A) was from Beyotime Biotechnology (Shanghai, China). 3, 3′-Dioctadecyloxacarbocyanine perchlorate (DIO, Cat. 40725ES10) was from YEASEN (Shanghai, China). Caspase-3 inhibitors (Ac-DMPD-CMK [DMPD] and Ac-DMLD-CMK [DMLD]) were courtesy of Professor Li-Juan Cao (China Pharmaceutical University, Nanjing, China). DOX and 5-Aza-CdR were dissolved in phosphate-buffered saline (PBS, Servicebio, Cat. G0002-2L) and stored at −20°C. SIM, DIO, and the caspase-3 inhibitors were dissolved in dimethyl sulfoxide (DMSO, Sigma, Cat. D2650) and stored at −20°C.

### Cell Treatment

Cells were seeded in 10 cm-diameter culture plates. Once the cells were 80% confluent, they were treated with certain concentration of SIM or with equal volumes of DMSO as control, for 48 h. For the inhibitor experiments, DMPD (5 μM) or DMLD (5 μM) was added to the cells 6 h before SIM treatment. For the SIM and 5-Aza-CdR combination experiments, MGC-803 and HGC-27 cells were incubated in medium containing 1 μM 5-Aza-CdR, refreshed daily for 72 h (Akino et al., 2007; Yanokura et al., 2020) before treatmen with 5 μM SIM (MGC-803 cells) or 20 μM SIM (HGC-27 cells) in the new medium for another 48 h.

### Cell Viability Assay

Cell proliferation was measured by a Cell Counting Kit-8 (CCK8) Assay Kit following manufacturer’s instructions (YEASEN, Cat. 40203ES60). The data is presented as the percent (%) of viable cells relative to the control.

### Lactate Dehydrogenase Release Assay

Cellular toxicity was detected using an LDH Cytotoxicity Assay kit, following the manufacturer’s instructions (Beyotime Biotechnology, Cat. C0017). Cellular membrane integrity was evaluated by the amount of LDH leaking from the damaged cell membrane and presented as fold changes to the control.

### Transmission Electron Microscopy (TEM)

MGC-803 cells were treated with SIM (5 μM) and after 48 h incubation, they were harvested and fixed in 2.5% glutaraldehyde in PBS (pH 7.4) at 4°C, and post-fixed with 1.3% osmic tetroxide. Subsequently, the cells were dehydrated in graded alcohols, transferred to propylene oxide, and embedded in epoxy resin. Thereafter, ultrathin sections were stained with uranyl acetate followed by lead citrate. Finally, sections were transferred to copper grids and observed with an 80-kV transmission electron microscope (Wang et al., 2021).

### Plasmids Lentivirus Transfection

All plasmids (Tet-on overexpression GSDME plasmid, the plasmids of GSDME-shRNA and caspase 3-shRNA knockdown and their control plasmids) were constructed by Public Protein/Plasmid Library. The procedure for lentivirus transfection has already been described previously (Zhou et al., 2019). For GSDME gene expression induction, 1 μg/ml of DOX was used in the medium for 4 days incubation (Croft et al., 2011). Stable low-expression or over-expression of GSDME in MGC-803 cells and HGC-27 cells were detected by real-time quantitative PCR (RT-qPCR) and Western blotting.

### RNA Extraction and Real-Time quantitative PCR

Total RNA was extracted from the frozen tissue or cultured cells with TRIzol (Invitrogen, Cat. 15596018), following the manufacturer’s instructions. Expression of the target genes was quantified with reverse transcription and RT-qPCR kits (Takara, Cat. DRR820A), normalized to the housekeeping gene β-actin. The relative gene expression levels were determined by the comparative threshold cycle (2^−ΔΔCT^) method. Supplementary Table S1 shows the primers used in the RT-qPCR.

### Western Blotting

Protein lysates were processed in RIPA-buffer (Beyotime, Cat. P0013C) supplemented with phosphatase inhibitor cocktail (Beyotime, Cat. P1050) and protease inhibitors (Beyotime, Cat. P1010). The protein was quantified by bicinchoninic acid (BCA) analysis (Beyotime, Cat. P0012). The protein extracts (30 μg/lane) were separated on SDS-PAGE gels, followed by electrotransferring onto polyvinylidene fluoride (PVDF) membranes. After blocking with 5% fat-free milk for 2 h at room temperature, the membranes were incubated with primary antibodies and subsequently incubated with secondary antibody. Information regarding the primary antibodies used is listed in Supplementary Table S3.

### Colony Formation Assay

The cells (5000 cells/well) were seeded into 6-well plates. After overnight incubation, they were subjected to the indicated treatment and then cultured in a fresh medium for 2 weeks. Colonies were fixed in 4% paraformaldehyde and visualized with 0.1% crystalline violet.

### Methylation Analyses

The association between GC and other common cancers with the degree of GSDME gene methylation was assessed using DiseaseMeth version 2.0 (http://biobigdata.hrbmu.edu.cn/diseasemeth/, accessed 30 August 2021). The database is used to provide information on abnormal DNA methylation in human diseases, especially cancer, in the most complete collection and annotations to date (Xiong et al., 2017).

### Methylation-Specific PCR

The genomic DNA was extracted using a genomic DNA kit (Tiangen Biotech, Beijing, China) to determine the GSDME methylation status by MSP. Briefly, genomic DNA was bisulfite-treated using an EZ DNA Methylation-Gold Kit (Zymo Research, Cat. D5005). Bisulphite-treated DNA was amplified using primers specific for either methylated or unmethylated DNA, designed by Sangon Biotech (Shanghai, China). The methylated DNA-specific (M) and unmethylated DNA-specific (U) primer sequences for GSDME are shown in Supplementary Table S5.

### Pyroptotic Cell Imaging

To observe the features of pyroptosis, the cells were plated in 24-well plates. The cytomembrane and the nucleus of cells post-treatment were stained with DIO (green fluorescence) and Hoechst 33342 (blue fluorescence), respectively. Fluorescence images were photographed using an inverted fluorescence microscope (Nikon Eclipse Ti, Tokyo, Japan).

### *In Vivo* Experiments

All procedures involving animals were reviewed and approved by the Guizhou Medical University the Animal Care Welfare Committee (approval number: 2001338). Five-week-old female immunodeficient BALB/c nude mice were purchased from the SLRC Laboratory Animal Center (Shanghai, China). MGC-803 cells w/wo Tet-on system for DOX-indicible GSDME were implanted subcutaneously in the right flanks of the mice to study the antitumor effects of SIM w/wo GSDME overexpression (mouse set 1) and w/wo the auxiliary therapy of 5-Aza-CdR (mouse set 2), respectively. When the tumours reached a volume of approximately 60 mm^3^, the mice were randomly divided into four groups (n = 5 each). Groups in set 1 were DOX (-) vehicle control, DOX (+) GSDME overexpression, DOX (-) with SIM, and DOX (+) with SIM, as summarized in Supplementary Figure S1A. Groups in set 2 were vehicle control, 5-Aza-CdR, SIM and SIM+5-Aza-CdR group, as illustrated in Supplementary Figure S1B. Mice were weighed every 2–3 days. The tumour size was measured with digital callipers every 2 or 3 days. Tumour volume was calculated using the following equation: volume = (width^2^ × length)/2. In each group, changes in the tumor sites were recorded every 7 days by the camera. The treatment lasted 18 days. At the end of the experiment, blood, tumours, livers, lungs, hearts, spleens, and kidneys of the mice were collected for subsequent experiments. Half of the tumour tissues and main organs were fixed in formalin and were paraffin-embedded for immunohistochemical (IHC) analysis, haematoxylin and eosin (H&E) staining and terminal deoxynucleotidyl transferase dUTP nick end labelling (TUNEL) assay (Roche, Cat. 1215679910). After TUNEL staining, the nucleus was counterstained with DAPI. The TUNEL-positive cells were photographed using an Ortho-Fluorescent Microscope (Nikon, Tokyo, Japan). The remaining tumour tissues were quickly frozen in liquid nitrogen and stored at −80°C for protein and nucleic acid detection. The blood was placed at room temperature for 2 h, centrifuged at 1,000 rpm for 20 min, and the separated serum was frozen at −80°C for subsequent experiments. The serum aspartate transaminase (AST), alanine transaminase (ALT), blood urea nitrogen (BUN), and creatinine (CRE) levels were analysed with an automated biochemical analyser, Chemray 240 (Lei Du Life Scientific and Technical Corporation, Shenzhen, China).

### H&E Staining and Immunohistochemical

Xenograft tumour tissues and main organs of the mice as well as human GC tissues and paired adjacent non-cancerous tissues were dewaxed in xylene and rehydrated in a graded alcohol series. Tumour tissue and main organ morphology were observed using H&E staining. IHC was performed using antibodies against Ki67, GSDME, and cleaved (Cl)-caspase-3 using an immunohistochemical test kit (Boster, SA1020). Images were captured using a Nikon E100 upright microscope (Nikon, Tokyo, Japan). The source and dilution of the primary antibodies are listed in Supplemenatry Table S2. The mean analyses of integrated optical density (IOD) in IHC staining were calculated with Image-ProPlus6.0 (Media Cybernetics, Silver Spring, MD, United States). Protein expression levels were quantified by IOD in five random visual fields (×40) before calculating the average value. For animal experiments, from each group of mice, tissues of three mice were selected for statistical analysis.

### Statistical Analysis

GraphPad Prism 7.04 (GraphPad, San Diego, CA, United States) was used for statistical analysis and graphics. The data are the mean ± SD of at least three independent experiments. Comparisons between two groups were analyzed by Student’s t-test. Comparisons between multiple groups were made using one-way analysis of variance (ANOVA), and two-way ANOVA for comparisons between multiple groups with independent variables. A *p*-value < 0.05 was considered significant.

## Results

### SIM Activates GSDME-Dependent Pyroptosis in GC Cells

**FIGURE 1:**
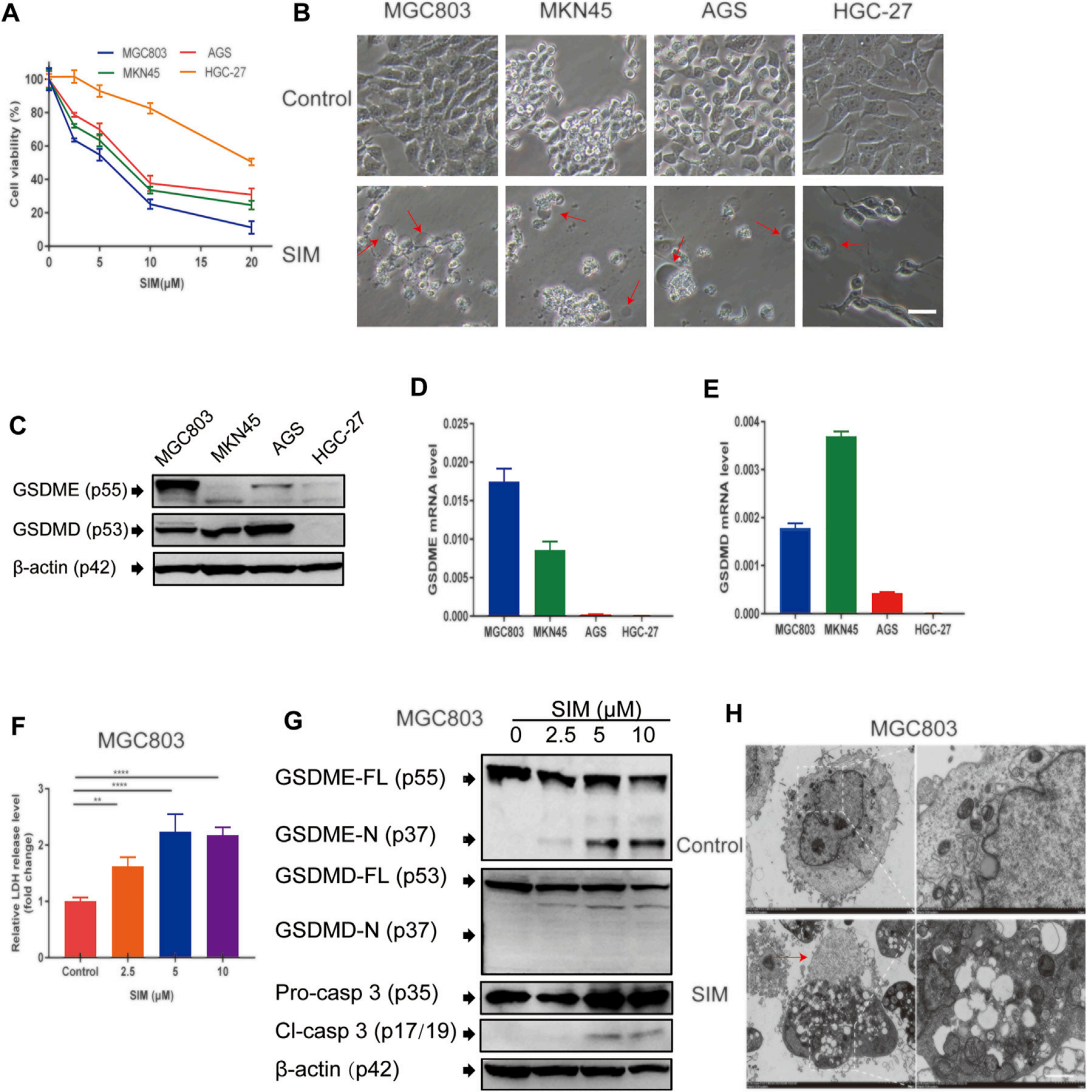
SIM induces GSDME-dependent pyroptosis in GC cells**. (A)** Cell viability of four GC cell lines treated with different doses of SIM for 48 h, detected by CCK8 (*n* = 3). **(B)** Microscopic imaging of four GC cell lines treated with SIM. Red arrows indicate ballooned cell membrane, a characteristic of pyroptotic cells; scale bar = 50 μm. **(C)** Protein levels of GSDME and GSDMD in four GC cell lines, determined by Western blotting. **(D,E)** The mRNA expression of GSDME and GSDMD gene, detected by RT-qPCR. **(F)** Release of LDH into the culture supernatant, detected by LDH assay kit (*n* = 4). **(G)** Protein levels of full length (FL) or N-terminal of GSDME and GSDMD, pro-caspase 3 (pro-casp3) and cleaved caspase-3 (Cl-casp3) of SIM-treated MGC-803 cells. **(H)** Electron microscopic images of MGC-803 cells treated with 5 μM SIM for 48 h. Red arrows indicate ballooned cell membrane characteristic of pyroptotic cells; scale bar = 10 μm. Data are shown as mean ± SD or representatives of at least three independent experiments. β-actin was used as an internal control for Western blot. ^∗∗^ *p* < 0.01, and ^∗∗∗∗^ *p* < 0.0001.

Cell proliferation of the four different GC cell lines was inhibited to various degrees by treatment of SIM from 2.5 to 20 μM for 48 h (Figure 1A). Among them, MGC-803 cells were the most sensitive (IC_50_ = 4.623 μM), while HGC-27 cells were the most resistant (IC_50_ = 20.24 μM) to SIM (Figure 1A; Table 1). Consistently, membrane ballooning, a signature of pyroptosis, was revealed extensively in SIM-treated MGC-803 cells by microscopy, but sparse in HGC-27 cells (Figure 1B). The basal expression levels of GSDME protein and mRNA were low or undetectable in AGS and HGC-27 cells (Figures 1C,D), whereas the MGC-803 cells had the highest GSDME expression at both mRNA and protein levels. The different abundance of GSDME in the four cell lines displayed a positive correlation with cell sensitivity to SIM, which was not shown in GSDMD expression (Figures 1C,E). A dose-dependent increase in the LDH release rates in the culture medium of SIM-treated MGC-803 cells was detected (Figure 1F). SIM also induced the cleavage of caspase-3 (Cl-casp3) and GSDME (GSDME-N) in the MGC-803 cells in a dose-dependent manner, but not GSDMD (Figure 1G). Therefore, the involvement of GSDMD in SIM-induced pyroptosis is disproved. Treatment of SIM at 5 μM was chosen for all subsequent experiments in MGC-803 cells as the cells would retain half of the viability, plateaued LDH release and significant GSDME-N cleavage, representing an ideal induction of pyroptosis. Further observation of the morphological alterations by TEM showed that SIM-treated cells had typical pyroptosis features including membrane pore formation, membrane leakiness and organelle swelling, and low cytosol density (Figure 1H), confirming the microscopy results (Figure 1B). Collectively, these data indicate that SIM induces pyroptosis in GC cells by activating GSDME.

**TABLE 1:** IC_50_ values of SIM treatment on four GC cell lines.

Cell lines	IC_50_ (μM)	±SD
MGC-803	4.623	0.030
MKN45	6.630	0.026
AGS	8.376	0.033
HGC-27	20.240	0.016

SD, standard deviation.

### SIM-Induced Pyroptosis in GC cells Involves Caspase-3-Mediated Cleavage of GSDME

**FIGURE 2:**
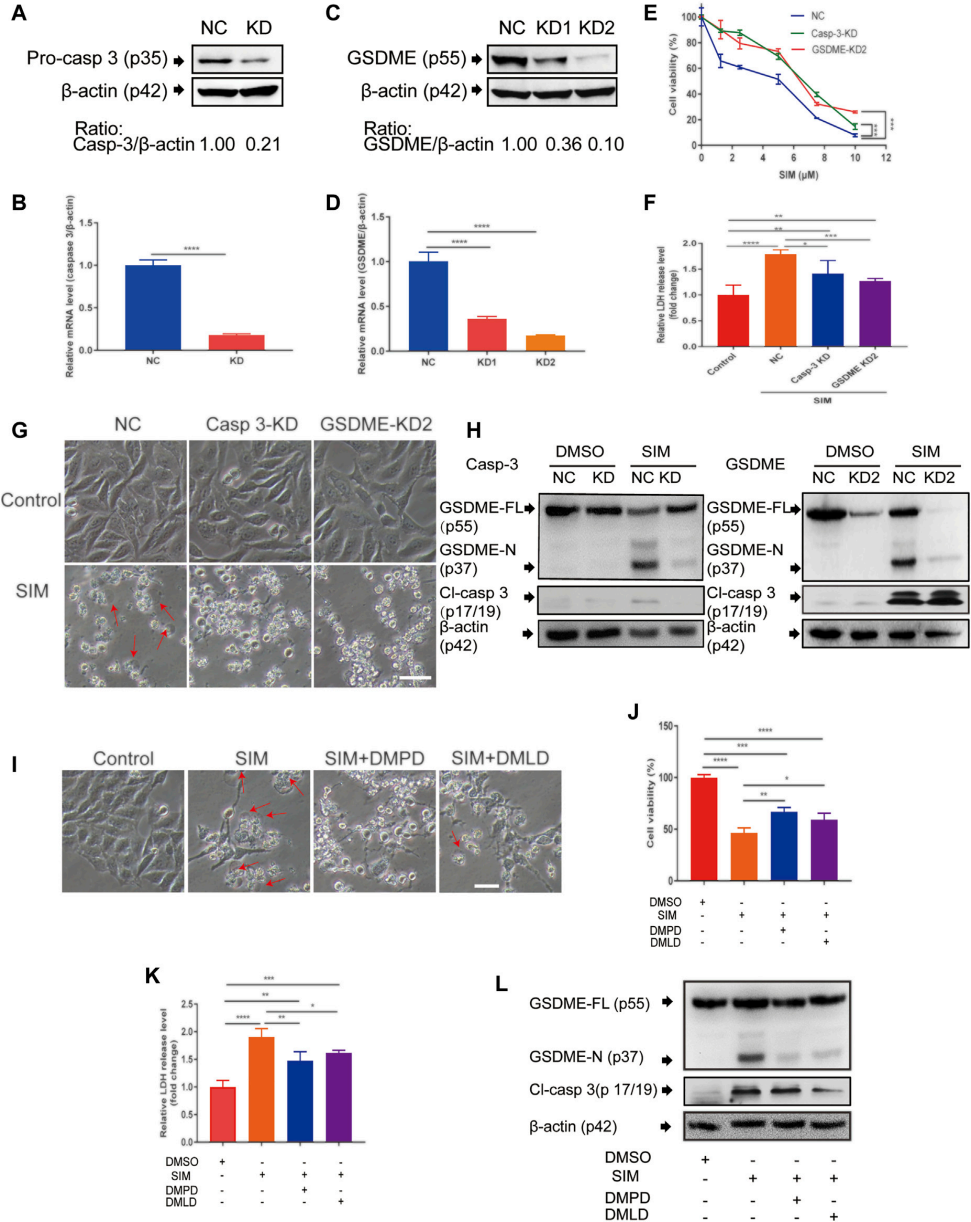
Caspase-3 and GSDME are required in SIM-induced pyroptosis. **(A–D)** Validation of the shRNA knockdown of caspase 3 or GSDME in MGC-803 cells by Western blotting and RT-qPCR. After the treatment of SIM at concentrations ranging from 0 to 10 μM with caspase 3 or GSDME knockdown, scrambled NC as the control, **(E)** cell viability was detected by CCK8 (*n* = 3) and **(F)** release of LDH into the culture supernatant was detected by LDH assay kit (*n* = 4), **(G)** Microscopic imaging of the cells treated with 5 μM SIM for 48 h, w/wo caspase 3 or GSDME knockdown. Red arrows indicate ballooned cell membrane; scale bar = 50 μm (*n* = 3). **(H)** Representative immunoblotting analysis of GSDME and cl-casp3 of the cells treated with 5 μM SIM for 48 h, w/wo caspase 3 or GSDME knockdown. **(I–L)** Microscopic morphology, cell viability, LDH release and protein cleavage of GSDME and caspase-3 of MGC-803 cells treated with 5 μM SIM for 48 h, w/wo the caspase-3 inhibitor Ac-DMPD-CMK (DMPD) or Ac-DMLD-CMKR (DMLD). Data are shown as mean ± SD or representatives of at least three independent experiments. β-actin was used as an internal control for Western blot. ^∗^ *p* < 0.05, ^∗∗^ *p* < 0.01, ^∗∗∗^ *p* < 0.001 and ^∗∗∗∗^ *p* < 0.0001.

In MGC-803 cells with stable knock down of caspase-3 (CASP3) or GSDME by lentiviral shRNA (Figures 2A–D), SIM-induced proliferation inhibition (Figure 2E), LDH release (Figure 2F), cell swelling (Figure 2G), cleavage of GSDME-N and Cl-caspase 3 (Figure 2H) were all significantly blunted. Similar blocking effects were observed in the cells treated with caspase-3 inhibitor DMPD or DMLD, 6 h prior to SIM administration (Figures 2I–K). DMPD and DMLD bind directly to the catalytic domains of caspase-3 to specifically inhibit the activity of GSDME. The results indicated that caspase-3 and GSDME are necessary for SIM-induced pyroptosis.

### GSDME Overexpression Enhances SIM-Induced GC Cell Pyroptosis

**FIGURE 3:**
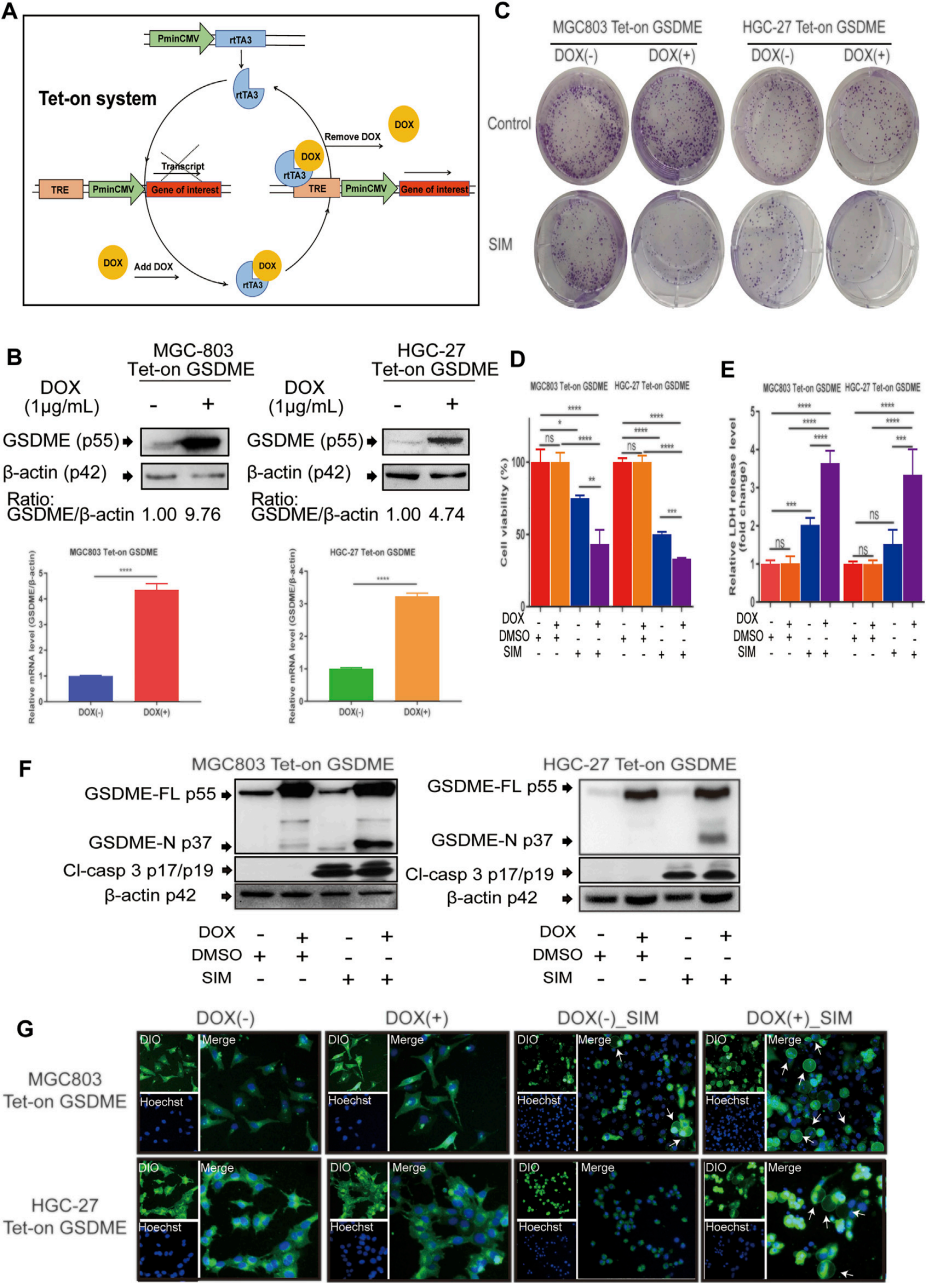
Overexpression of GSDME enhanced SIM-induced cell pyroptosis. **(A)** Schematic representation of the DOX- controlled Tet-on system. **(B)** Validated of GSDME overexpression in MGC-803 or HGC-27 cells by Western blotting and RT-qPCR. Colony formation **(C)**, Cell viability **(D)**, LDH release **(E)** and protein cleavage of GSDME and caspase-3 **(F)** of GSDME-overexpressing MGC-803 and HGC-27 cells treated with SIM for 48 h. **(G)** Fluorescence microscopy imaging of GSDME-overexpressing MGC-803 and HGC-27 cells treated with SIM for 48 h, DIO (green fluorescence) was used to dye cell cytomembrane, and Hoechst 33342 (Blue fluorescence) the cell nucleus. White arrows indicate ballooned cell membrane; scale bar = 50 μm. Data are shown as mean ± SD or representatives of at least three independent experiments. β-actin was used as an internal control for Western blot. ^∗^ *p* < 0.05, ^∗∗^ *p* < 0.01, ^∗∗∗^ *p* < 0.001 and ^∗∗∗∗^ *p* < 0.0001.

To manipulate the gene expression of GSDME, we used the doxycycline (DOX)- inducible Tet-on system that allows reversibly switching on or off the GSDME gene by DOX (Figure 3A). The efficiency of the system was confirmed by successful induction of GSDME overexpression in MGC-803 and HGC-27 cells upon the addition of DOX to the culture medium (Figure 3B). While adding DOX alone had no impact on cell viability and colony formation, it synergistically enhanced the SIM-induced inhibition of proliferation and clonogenicity of these cells (Figures 3C,D). Moreover, LDH release (Figure 3E), GSDME cleavage (Figure 3F) and cell swelling (Figure 3G) were augmented with consistency, demonstrating an additive effect of GSDME overexpression on SIM-induced pyroptosis.

### Combining SIM With GSDME Overexpression Potently Inhibits Tumour Growth *In Vivo*

**FIGURE 4:**
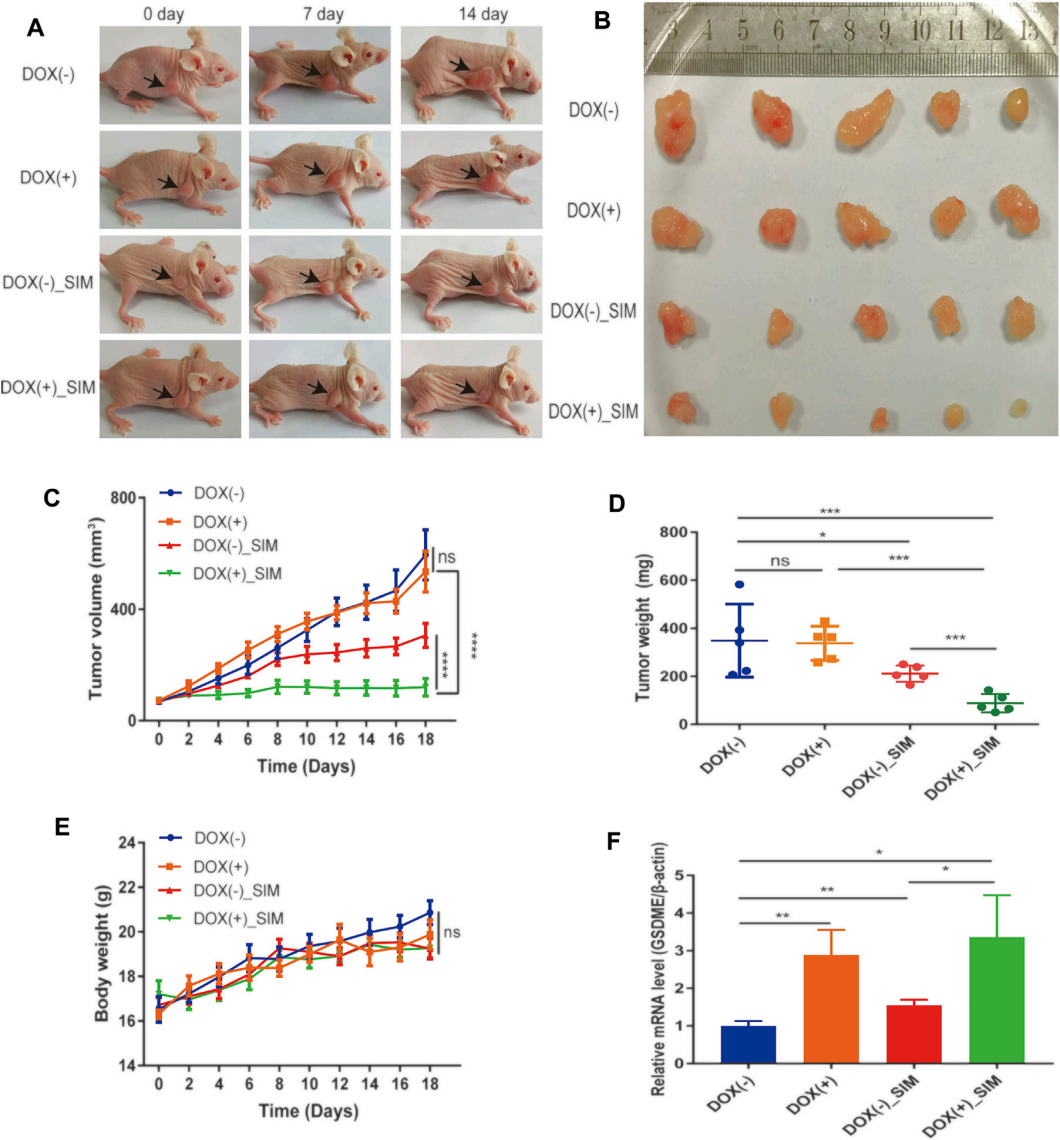
SIM and GSDME overexpression additively inhibit tumour growth in mouse xenografts. **(A)** Photos of mice with xenograft tumours during the treatment. **(B)** Tumours dissected and imaged at the end point of experiment. **(C)** Tumour volumes during the treatment (*n* = 5). **(D)** Tumour weights at the end of the experiment (*n* = 5). **(E)** Average body weight of each group (*n* = 5) **(F)** The of GSDME gene expression in xenograft tumour tissues, detected by RT-qPCR (*n* = 3). ^∗^ *p* < 0.05, ^∗∗^ *p* < 0.01, ^∗∗∗^ *p* < 0.001, ^∗∗∗∗^ *p* < 0.0001 and *p* > 0.05 not significant (ns).

**FIGURE 5:**
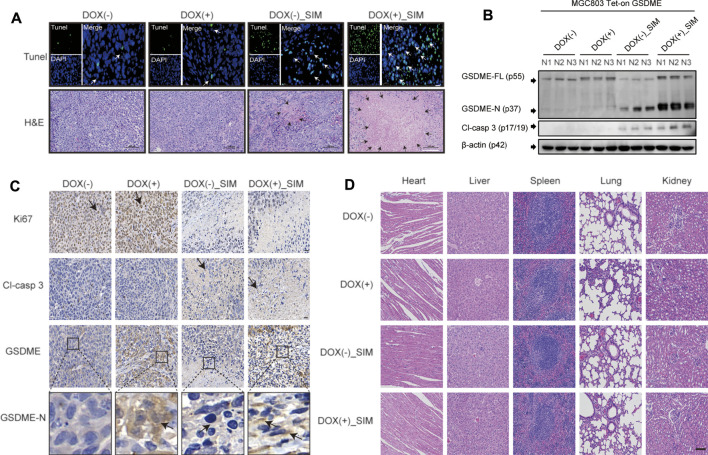
SIM combined with overexpression-GSDME promotes the progression of GC pyroptosis *in vivo*. **(A)** TUNEL and H&E staining images for tumour slices after indicated treatment. White arrows indicate TUNEL positive cells. TUNEL scale bar: 20 μm; the areas of necrosis were indicated by black arrows in the figures. H&E scale bar: 100 µm. **(B)** Protein cleavage of GSDME and caspase-3 in tumour tissues, detected by Western blotting **(C)** Immunohistochemistry (IHC) images of Ki67, Cl-casp3, GSDME and GSDME-N for tumour tissues after indicated treatment; black arrows indicate the IHC staining-positive cells; scale bar: 20 µm. **(D)** HE staining of heart, liver, spleen, lung, and kidney tissues in each group; scale bar = 100 µm.

To examine the *in vivo* GC tumorigenicity under influence of GSDME regulation, we established a xenograft model by injecting the DOX-inducible Tet-on MGC-803 cells into nude mice (Supplementary Figure S1A). During the 18-day treatment, the changes at tumour sites of each group were recorded every 7 days (Figure 4A). Both SIM group and DOX (+) _SIM group demonstrated significantly inhibited tumour growth (Figure 4B) and reduced tumour volume (Figure 4C) and tumour weight (Figure 4D), but no impact on body weight (Figure 4E). Compared to SIM alone, the extent of the inhibition was notably exaggerated when GSDME gene expression was promoted in the xenograft tissues by intake of DOX water by the mice (Figures 4A–F), whereas switching on the GSDME gene expression *per se* did not affect tumour development (Figures 4C–E). H&E and TUNEL staining indicated that GSDME overexpression combined with SIM induced more intensive DNA fragmentation and ultimately cell death in the tumour (Figure 5A), accompanied by decreased Ki67 expression, a cell proliferation marker (Figure 5C; Supplementary Figure S2A). The increased Cl-caspase-3 and GSDME-N abundance in the SIM group were evidently boosted by GSDME overexpression in DOX (+) SIM group, shown consistently by western blot (Figure 5B) and IHC (Figure 5C; Supplementary Figures S2B,C). Meanwhile, no damage was observed in the heart, spleen, lung, and kidney, compared between all groups (Figure 5D). The functionality of the liver and kidney remained intact, as suggested by the healthy serum levels of AST, ALT, BUN, and CRE in all groups (*p* > 0.05) (Supplementary Table S5). These *in vivo* data confirmed the previous finding that GSDME overexpression enhanced SIM-induced cell growth inhibition and pyroptosis by activating the caspase-3/GSDME pathway.

### GSDME Demethylation Enhances SIM-Induced GC Cell Pyroptosis

**FIGURE 6:**
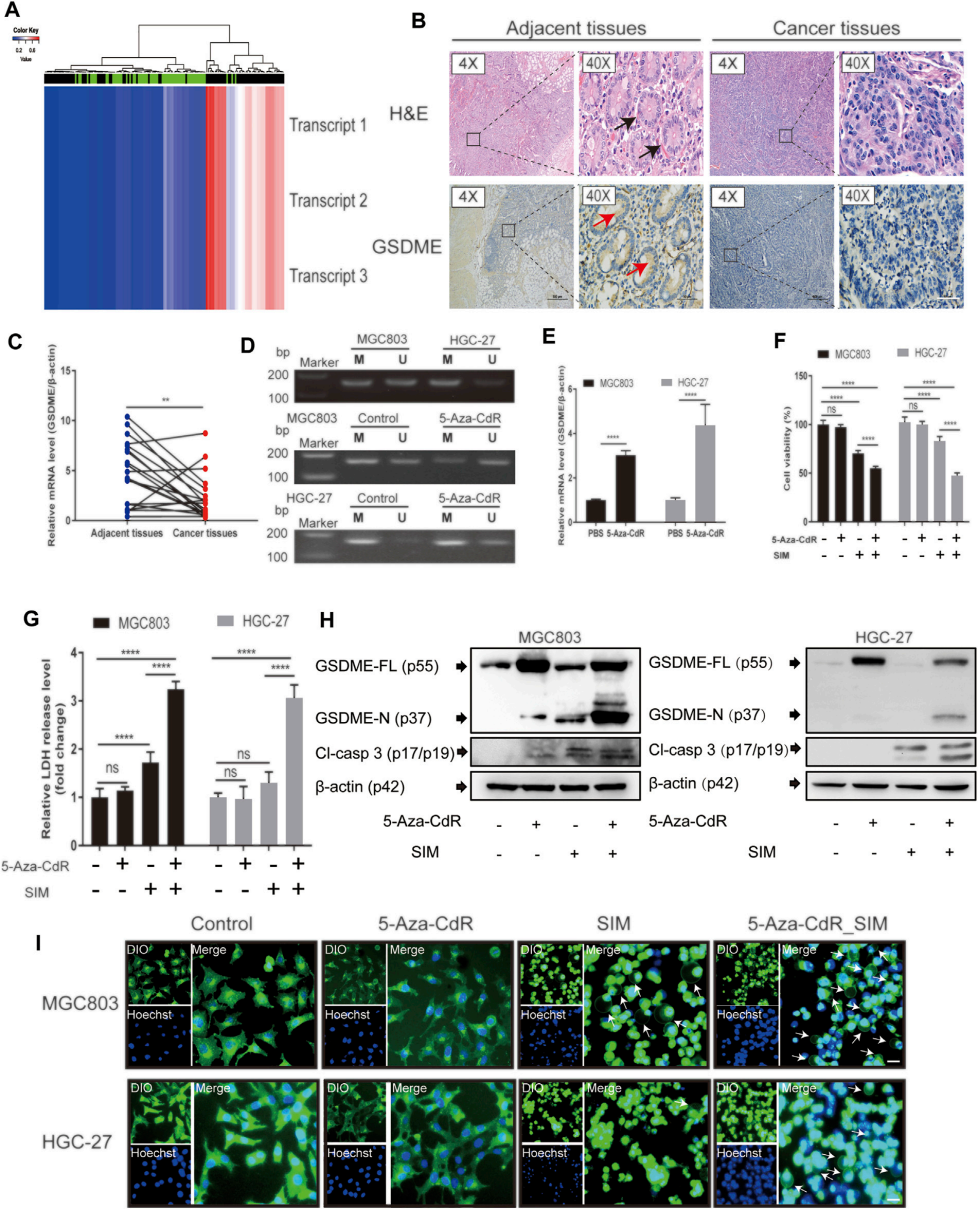
Combination of SIM and 5-Aza-CdR enhances pyroptosis in GC. **(A)** The heatmap of GSDME methylation in GC. The heatmap contains methylation data of 3 transcripts from 141 samples of 27 k arrays. Rows represent transcripts and columns represent samples (green: normal profiles, black: disease profiles). **(B)** GSDME protein levels in human GC tissues and adjacent tissues, detected immunohistochemically using HE staining; black arrows indicate complete gastric gland; red arrows indicate the IHC staining-positive cells; scale bar = 50 µm. **(C)** Relative GSDME mRNA expression in human GC tissues and adjacent normal tissues, determined by RT-qPCR (*n* = 20). **(D)** MSP profiles of GSDME promoter methylation status in MGC-803 and HGC-27 cell before and after a daily dosing of 5-Aza-CdR for 72 h, M, methylated; U, unmethylated. After SIM treatment w/wo 5-Aza-CdR pre-dosing, **(E)** GSDME gene, **(F)** Cell viability, **(G)** LDH release, **(H)** protein cleavage of GSDME and caspase-3, and **(I)** fluorescence microscopy of theMGC-803 and HGC-27 cells examined. Data are shown as mean ± SD or representatives of at least three independent experiments. β-actin was used as an internal control for Western blot. ^∗∗∗∗^ *p* < 0.0001 and *p* > 0.05; ns, not significant.

The association between some common cancers and GSDME gene methylation was examined using DiseaseMeth version 2.0. Among all the malignancies examined, GC had the highest statistical significance of its positive association with GSDME hypermethylation (*p* = 1.62E-12, Table 2). The heatmap of methylation showed that GC tissues had a significantly higher degree of GSDME methylation than the normal tissues (Figure 6A). In the GC tissues, GSDME protein and mRNA levels were downregulated compared with the adjacent normal tissues, as determined by IHC and RT-qPCR (Figures 6B,C; Supplementary Figure S2D). However, no significant correlation was found between the clinicopathological parameters and GSDME expression levels in GC tissue of 20 patients with GC (Supplementary Table S1). MSP revealed partial methylation of GSDME at the promoter region in HGC-27 cells and to a lesser extent in MGC-803 cells (Figure 6D), which matched the superior resilience of HGC-27 cells to pyroptosis (Figure 1). Treatment of 5-Aza-CdR on MGC-803 and HGC-27 cells demethylated the GSDME gene (Figure 6D) and restored its mRNA transcription (Figure 6E). Cells pre-treated with 1 μM 5-Aza-CdR for 72 h were more responsive to SIM treatment than SIM alone, demonstrated by cell viability (Figure 6F), LDH release (Figure 6G), GSDME/caspase-3 cleavage (Figure 6H) and cell swelling (Figure 6I). These results implied that GSDME demethylation facilitated SIM-induced GC cell proliferation inhibition and pyroptosis.

**TABLE 2:** Association between disease and methylation of GSDME/DFNA5.

Disease name	Gene symbol	Mean methyl disease	Mean methyl normal	*p*-value	Methyl-type
Gastric cancer	GSDME/DFNA5	0.235	0.092	1.62E-12^∗∗∗∗^	hyper-methyl
Breast neoplasms	GSDME/DFNA5	0.54	0.441	1.89E-05^∗∗∗∗^	—
Esophageal cancer esophageal squamous cell carcinoma without metastasis	GSDME/DFNA5	0.392	0.308	3.22E-02^∗^	—
Brain cancer ganglioneuroma	GSDME/DFNA5	0.03	0.022	1.98E-01	—
Lung cancer lung squamous cell carcinoma	GSDME/DFNA5	0.45	0.446	5.14E-01	—

*p <  0.05 and ∗∗∗∗*p* < 0.0001.

### 5-Aza-CdR Amplifies the Antitumor Effects of SIM in a Xenograft Model

**FIGURE 7:**
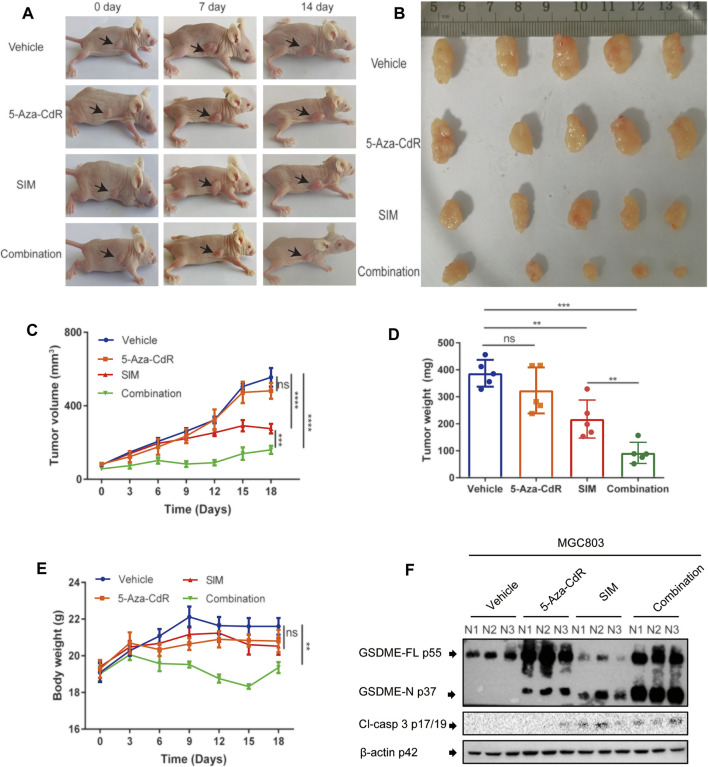
Combination of SIM and 5-Aza-CdR potently inhibits tumour growth *in vivo*. **(A)** Photos of mice with xenograft tumours during the treatment. **(B)** Tumours dissected at the end point of the experiment. **(C)** Tumour volumes during the treatment (*n* = 5). **(D)** Tumour weights at the end of the experiment (*n* = 5). **(E)** Average body weight of each group during the treatment (*n* = 5). **(F)** Protein cleavage of GSDME and caspase-3 in tumour tissues, detected by Western blotting (*n* = 3). Data are shown as mean ± SD or representatives of at least three independent experiments. β-actin was used as an internal control for Western blot. ^∗∗∗∗^ *p* < 0.0001 and *p* > 0.05 not significant (ns).

**FIGURE 8:**
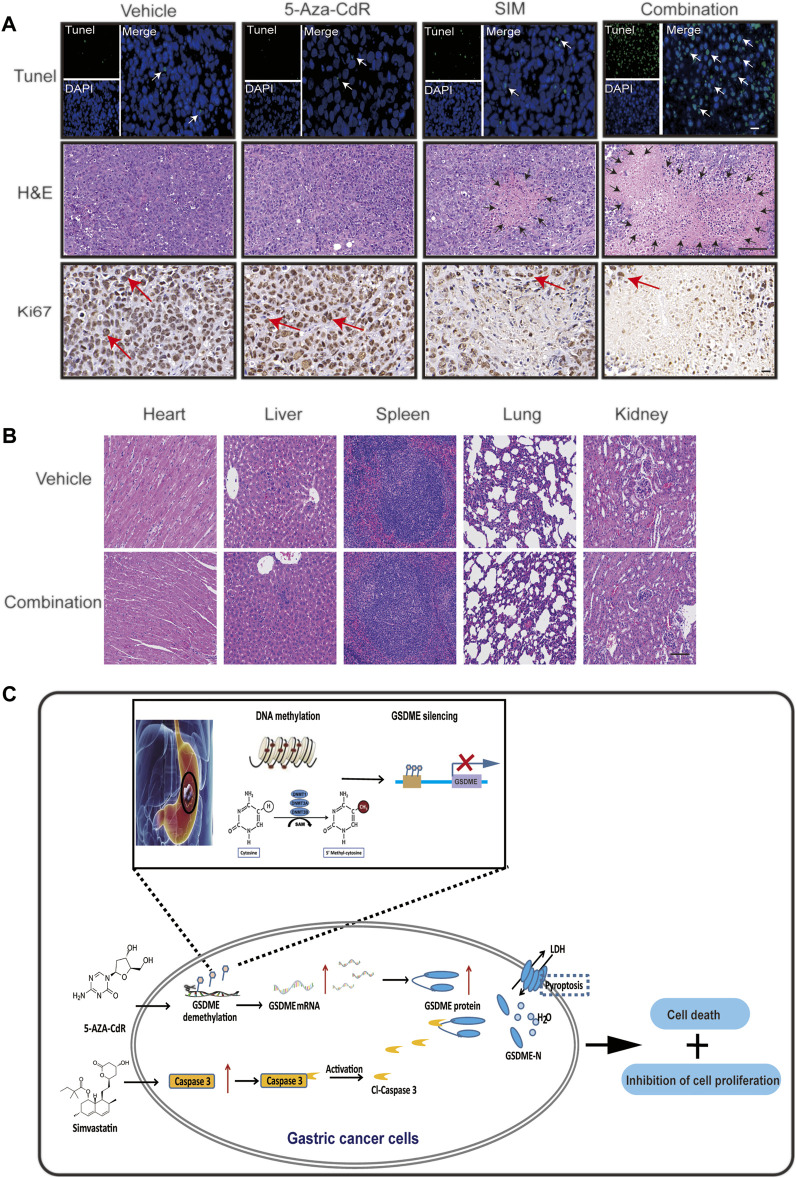
5-Aza-CdR promotes SIM-induced GC pyroptosis *via* caspase 3/GSDME pathway *in vivo*. **(A)** TUNEL, H&E staining and Ki67 IHC of xenograft tumour slices after indicated treatment. TUNEL scale bar: 20 µm. White arrows: TUNEL positive cells; H&E scale bar: 100 µm. Black arrows: the areas of necrosis; IHC scale bar: 20 µm. Red arrows: Ki67-positive cells. **(B)** HE staining of heart, liver, spleen, lung, and kidney tissues in each group; scale bar: 100 µm. **(C)** Schematic diagram to illustrate the mechanisms underlying the induction of pyroptosis in GC by a combined treatment of SIM with 5-Aza-CdR *via* caspase-3/GSDME activation.

Mice bearing GC xenograft was generated by subcutaneous transplantation of MGC-803 cells into nude mice (Supplementary Figure 1B). Compared to the vehicle group, both the SIM and SIM+5-Aza-CdR combination group had significantly inhibited tumour growth (Figures 7A,B) and reduced tumour volume (Figure 7C) and tumour weight (Figure 7D). The delay in tumour growth was considerably more pronounced in the combination group compared with the SIM group (Figures 7A–D), which mirrored what we observed in the Tet-on GSDME overexpression mouse model. However, rather than continuous and undistinguishable bodyweight gain in all groups, as seen in the previous xenograft experiment, the bodyweight of the mice in the SIM+5-Aza-CdR combination group decreased during treatment and started to catch up at day 15 (Figure 7E). Compared with the vehicle, 5-Aza-CdR monotherapy promoted GSDME expression (Figure 7F) but did not affect tumour growth (Figures 7C,D). Furthermore, compared with the SIM group, the combination group induced more significant caspase-3-dependent cleavage of GSDME (Figure 7F). As expected, TUNEL and H&E staining showed more remarkable DNA breakdown and diminished Ki67 staining in tumour tissues, reflecting an increased vulnerability of cancer cells to pyroptosis triggered by the combined therapy (Figure 8A) than the SIM monotherapy. In addition to that, there was no detectable damage found in the heart, liver, spleen, lung, and kidney by H&E staining (Figure 8B), and the serum concentrations of AST, ALT, BUN, and CRE remained in the normal range throughout all groups (Supplementary Table S6). In summary, GSDME demethylation enhanced SIM-induced GC cell proliferation inhibition and pyroptosis by regulating the caspase-3/GSDME pathway, without damage to the main organs.

## Discussion

Beyond cardiovascular protection *via* interrupting the biosynthesis of cholesterol, statins merit great attention for their antineoplastic activity (Jiang et al., 2021a), evidenced in multiple clinical observations and experimental investigations. A recent South Korean prospective study pointed out that the use of statins could reduce GC mortality in the general population (Cho et al., 2021). In particular, pleiotropic mechanisms have been proposed to underlie the action of SIM against cancers (Chiu et al., 2011), such as inhibiting tumour cell proliferation, migration and invasion, and promoting apoptosis by simultaneously targeting YAP and β-catenin signalling (Liu et al., 2020). Adding to the extant knowledge, we found that SIM treatment for GC induces pyroptosis by activating caspase-3 to cleave GSDME.

Both GSDMD and GSDME could perforate cell membrane to initiate the pyroptosis process (Yu and He, 2017; Shi et al., 2015). GSDMD is abundantly present in GC cell lines except HGC-27. However, we found that the IC50 value of SIM on CG cells was negatively correlated with the baseline expression level of GSDME, not GSDMD (Figure 1; Table 1). The signs of pyroptosis including plasma membrane swelling and LDH release in SIM-treated MGC-803 cells were accompanied by the generation of GSDME-N, not GSDMD-N (Figure 1). Blocking the caspase-3/GSDME pathway by shRNA knockdown or inhibitors attenuated the SIM-induced pyroptosis, while conversely overexpressing GSDME encouraged GC cells to shift into pyroptotic death. These data demonstrate that GSDME, not GSDMD, is responsible for the pyroptosis in GC cells induced by SIM, corroborating the previous reports that GSDMD-dependent pyroptosis occurs mainly in non-cancerous cells (Ding et al., 2021; Han et al., 2021; Shi et al., 2021) and GSDME-dependent pyroptosis in cancer cells (Cai et al., 2021; Li et al., 2021; Shangguan et al., 2021).

Targeting pyroptosis is considered a novel enrichment to our cancer-fighting arsenal (Guo et al., 2021; Yuan et al., 2021) and GSDME is the newly recognized executor of pyroptosis (Xia et al., 2020). It has been reported that dihydroartemisinin (DHA), a conventional drug to kill malaria parasites, can induce pyroptosis of esophageal squamous cell carcinoma (ESCC) cells *via* PKM2 caspase-8/3-GSDME pathway, providing a potential therapeutic agent for the treatment of ESCC (Jiang et al., 2021b). GSDME-dependent pyroptosis can also be triggered by chemotherapy (paclitaxel, cisplatin, lobaplatin, 5-fluorouracil (5-FU) and doxorubicin) or by targeted molecular drugs in malignant cells (Lu et al., 2018; Xia et al., 2020). As the GSDME gene is often silenced through hypermethylation in GC, either genetic tools or demethylating agents such as 5-Aza-CdR could restore the GSDME expression to facilitate a more efficient SIM therapy, as shown in our xenograft mice. As a potent methyltransferase inhibitor, 5-Aza-CdR is an FDA-approved epigenetic therapy to treat a wide array of cancers by reactivating multiple tumour suppressor genes (Christman, 2002). In our xenograft GC mice, combining SIM with GSDME overexpression demonstrated a tumour-specific targeting, and none of the main organs examined displayed observable damage. Nevertheless, effects of drug discontinuation and long-term administration, and potential adverse effects with a prolonged treatment duration, remain to be clarified by further investigation. Other vital tissues such as muscle and adipose would be included in future research too. In mice treated with both 5-Aza-CdR and SIM, we have seen a weight loss up to 37% to the initial value, which was rebounded promptly after treatment cessation. This side effect may be circumvented by an appropriate extension of the interval of the treatment cycle.

It must be stressed that SIM, the same as other statin medications, can exhibit anti-tumour effects from various aspects, such as metabolic reprogramming (Ni et al., 2021), immune regulation (Yu et al., 2022), and angiogenesis (Wesołowska et al., 2021), etc. that may not necessarily involve GSDME. Although the declined cell viability upon SIM treatment was attenuated significantly with disruption of caspase-3 or GSDME expression or function, the cytotoxicity of SIM was still profound, suggesting contributions of other mechanisms. How these different pathways crosstalk to each other is a burning question awaiting an answer.

In conclusion, SIM exerts an antitumor effect on GC *via* impaired cell proliferation and caspase-3/GSDME-mediated pyroptosis. Restorring GSDME expression by 5-Aza-CdR could be beneficial by sensitising GC cells to SIM.

## Limitations of the Study

We noticed that SIM increased the transcription of the GSDME gene in MGC-803 cells and in the xenograft (Figure 4F; Supplementary Figure S3), and had to leave it unaddressed in the present work. Interestingly, statin has been hinted at as a DNA methyltransferase (DNMT) inhibitor. By suppressing DNMT, statins such as lovastatin and simvastatin could upregulate a couple of genes that were silenced by promoter hypermethylation, including BMP2, in colorectal cancer cells and xenograft mice. The outcome of this modulation by statin was a reversal of the malignant cells from the stem-like state into a more differentiated state (Kodach et al., 2011). We may speculate a similar epigenetic mechanism operating in our models.

## Data Availability

The original contributions presented in the study are included in the article/Supplementary Material, further inquiries can be directed to the corresponding authors.
