# Supplementary material for: Antitumor Effect of Simvastatin in Combination With DNA Methyltransferase Inhibitor on Gastric Cancer via GSDME-Mediated Pyroptosis
Source: Front Pharmacol. 2022 Apr 20;13:860546. doi: 10.3389/fphar.2022.860546 (PMC9065610; doi:10.3389/fphar.2022.860546)
Supplement: Supplementary file 1 [file DataSheet2.PDF]

The raw data of this manuscript.

Figure 1

Figure 1B

Four GC cell lines were treated with SIM. The raw microscopy images were shown as below.

MGC803_Control

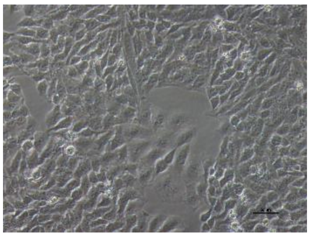

MGC803_SIM

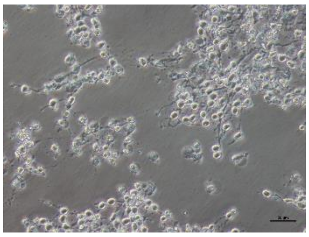

MKN45_Control

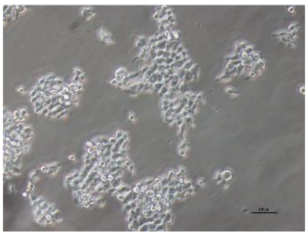

MKN45_SIM

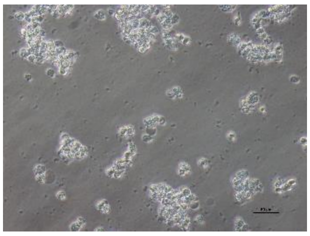

AGS_Control

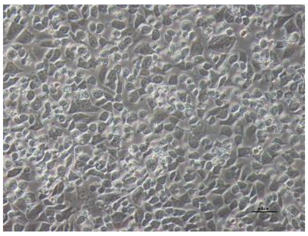

AGS_SIM

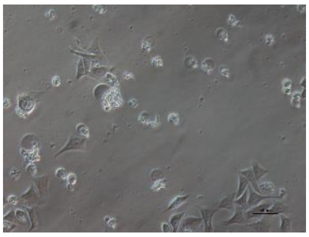

HGC-27_Control

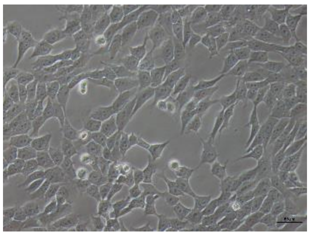

HGC-27_SIM

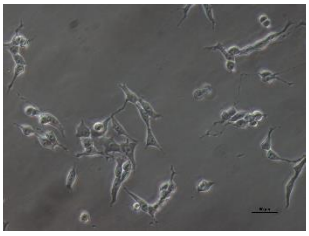

Figure 1C

The raw data were added protein names, grouping, and protein marker.

The protein expression of GSDME.

1 lane: marker；2 lane: GES-1; 3 lane: MGC803; 4 lane: MKN45; 5 lane: AGS; 6 lane: HGC-27.

*: nonspecific bands.

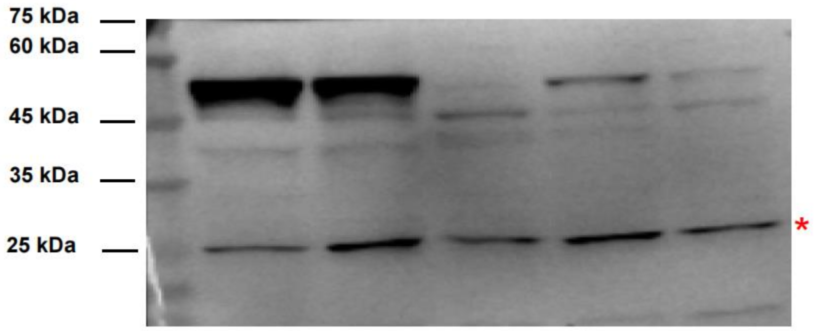

The protein expression of GSDMD.

1 lane: marker; 2 lane: GES-1; 3 lane: MGC803; 4 lane: MKN45; 5 lane: AGS; 6 lane: HGC-27.

*: nonspecific bands.

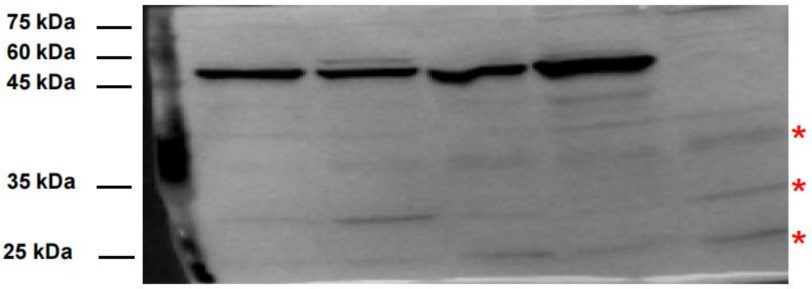

The protein expression of β-actin.

1 lane: marker; 2 lane: GES-1; 3 lane: MGC803; 4 lane: MKN45; 5 lane: AGS; 6 lane: HGC-27.

*: nonspecific bands.

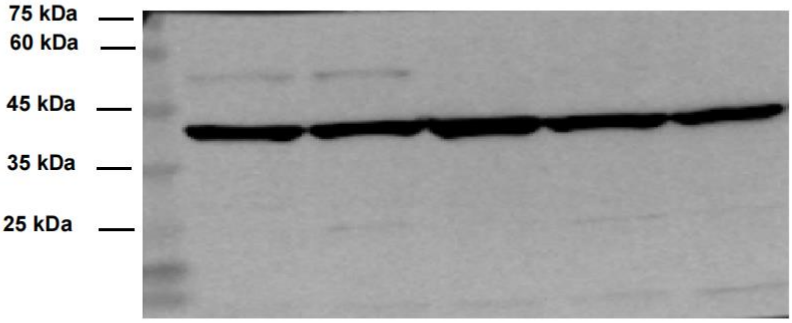

Figure 1G

The raw data were added protein names, grouping, and protein marker.

The protein expression of GSDME.

1 lane: marker; 2 lane: MGC803 Control; 3 lane: MGC803 SIM-2.5 μM; 4 lane:MGC803 SIM-5 μM; 5 lane: MGC803 SIM-10 μM.

*: nonspecific bands.

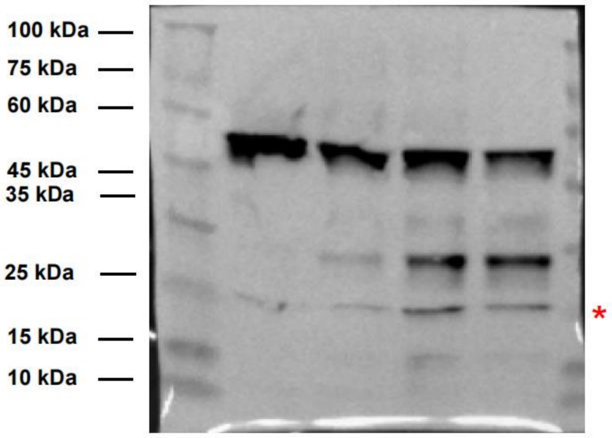

The protein expression of GSDMD.

1 lane: marker； 2 lane: MGC803 Control; 3 lane: MGC803 SIM-2.5 μM; 4 lane: MGC803 SIM-5 μM; 5 lane: MGC803 SIM-10 μM.

*: nonspecific bands.

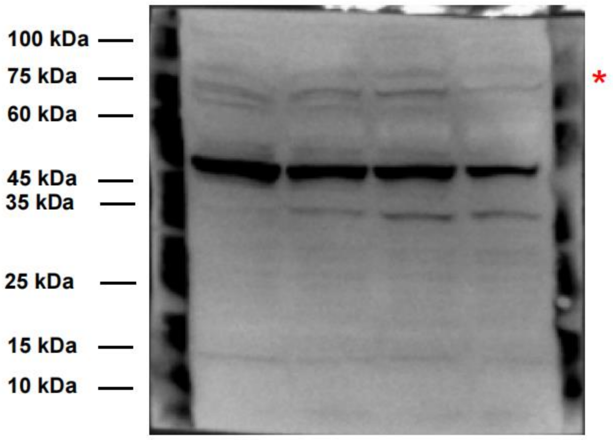

The protein expression of pro-casp 3 and cl-casp 3.

1 lane: marker； 2 lane: MGC803 Control; 3 lane: MGC803 SIM-2.5 μM; 4 lane: MGC803 SIM-5 μM; 5 lane: MGC803 SIM-10 μM.

*: nonspecific bands.

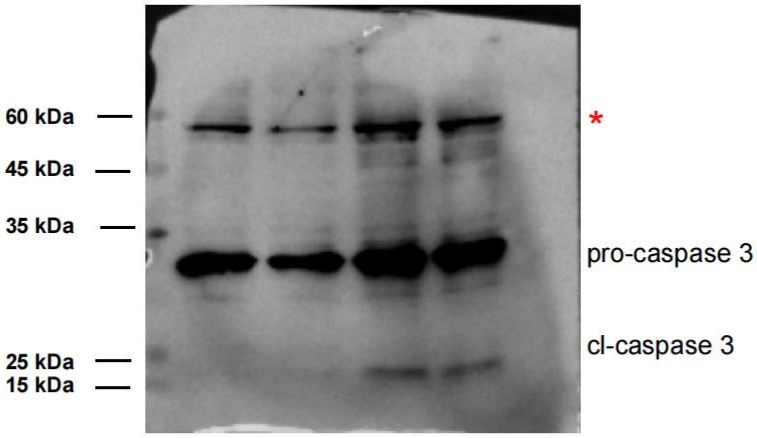

The protein expression of β-actin.

1 lane: marker； 2 lane: MGC803 Control; 3 lane: MGC803 SIM-2.5 μM; 4 lane: MGC803 SIM-5 μM; 5 lane: MGC803 SIM-10 μM.

*: nonspecific bands.

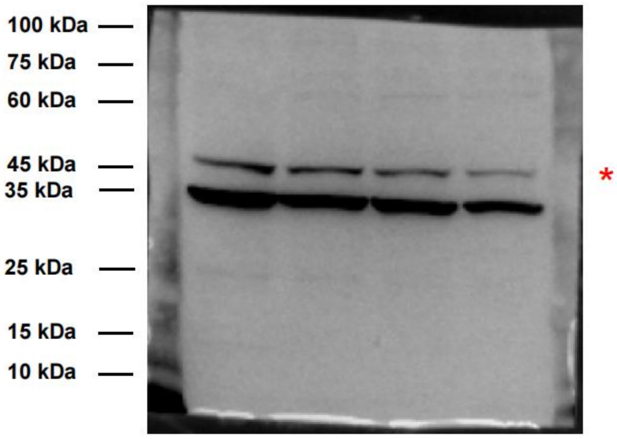

Figure 1 H

The picture of TEM

MGC803 Control (scale bar: 5 μm)

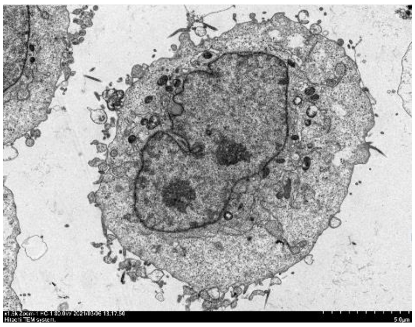

MGC803 Control (scale bar: 1 μm)

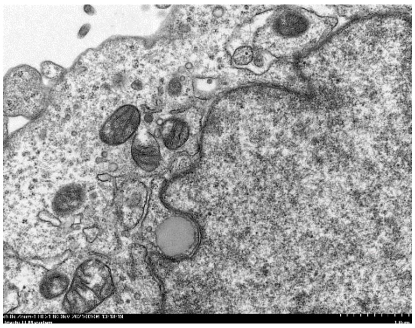

MGC803-SIM (scale bar: 5 μm)

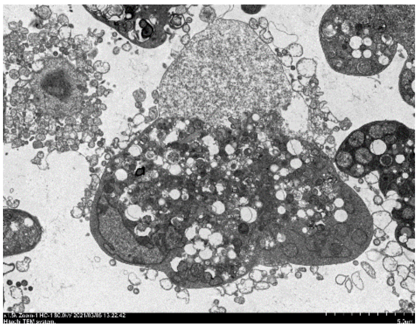

MGC803-SIM (scale bar: 1 μm)

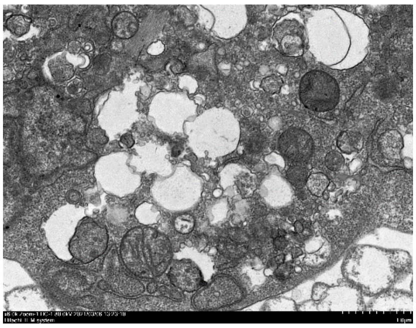

Figure 2

Figure 2A

The protein expression of pro-casp 3 and β-actin.

1 lane: marker； 2 lane: MGC803 sh-NC; 3 lane: MGC803 sh-casp 3

*: nonspecific bands.

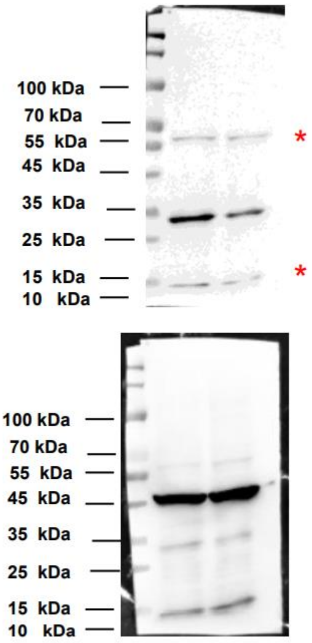

Figure 2C

The protein expression of GSDME and β-actin.

1 lane: marker；2 lane: MGC803 sh-NC; 3 lane: MGC803 sh-GSDME

*: nonspecific bands.

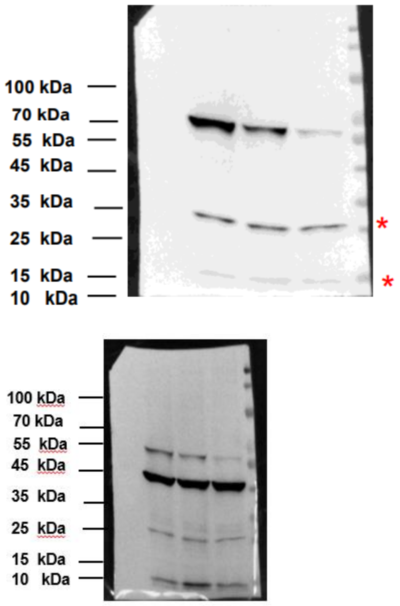

Figure 2G

MGC803_NC_Control MGC803_NC_SIM

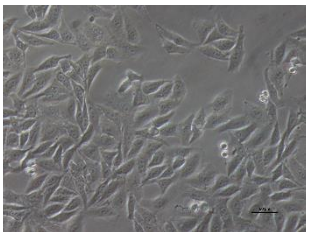
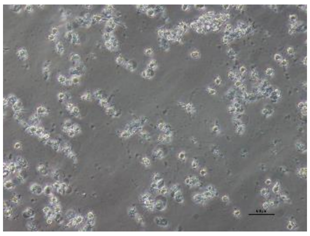

MGC803_Casp 3-KD_Control MGC803_Casp 3-KD_SIM

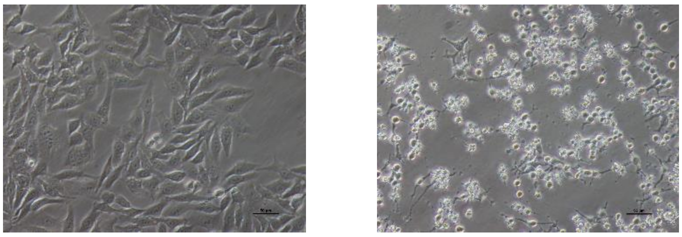

MGC803_GSDME-KD2_Control   MGC803_GSDME-KD2_SIM

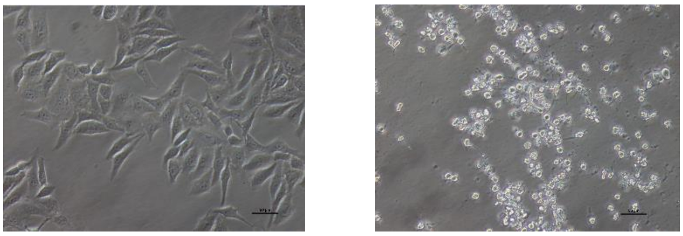

Figure 2H

The protein expression of GSDME, cl-casp3 and β-actin.

1 lane: marker； 2 lane: MGC803 sh-NC DMSO; 3 lane: MGC803 sh-casp 3 DMSO; 4 lane: MGC803 sh-NC SIM; 5 lane: MGC803 sh-casp 3 SIM

*: nonspecific bands.

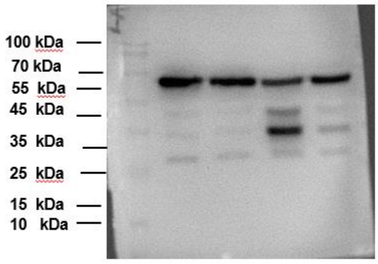

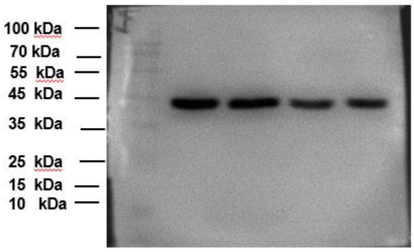

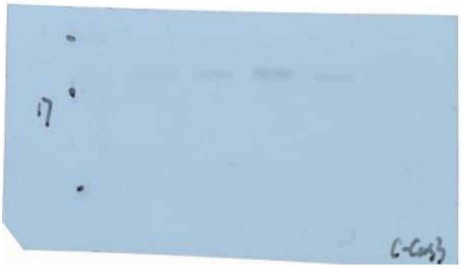

The protein expression of GSDME and β-actin.

1 lane: marker； 2 lane: MGC803 sh-NC DMSO; 3 lane: MGC803 sh-GSDME DMSO; 4 lane: MGC803 sh-NC SIM; 5 lane: MGC803 sh-GSDME SIM

*: nonspecific bands.

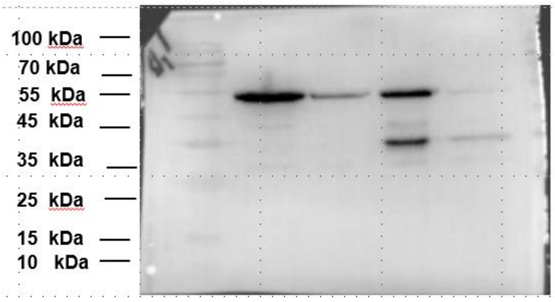

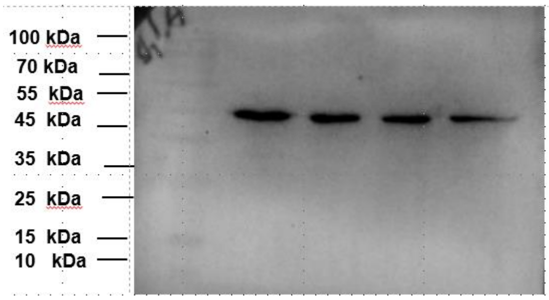

The protein expression of cl-casp3.

1 lane: marker； 2 lane: MGC803 sh-NC DMSO; 3 lane: MGC803 sh-GSDME DMSO; 4 lane: MGC803 sh-NC SIM; 5 lane: MGC803 sh-GSDME SIM

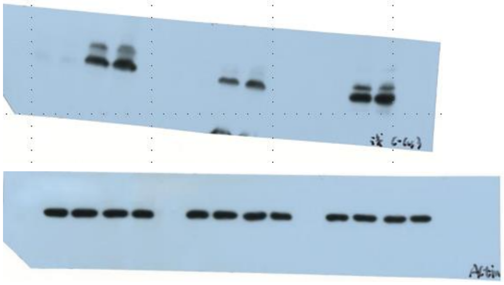

Figure 2I

MGC803_Control

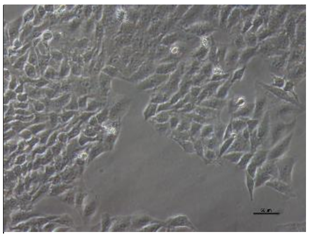

MGC803_SIM

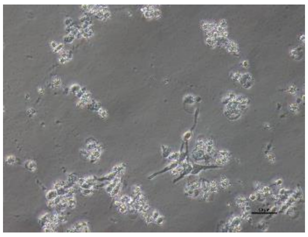

MGC803_SIM_DMPD

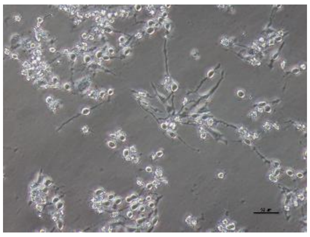

MGC803_SIM_DMLD

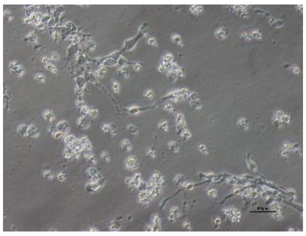

Figure 2N

The protein expression of GSDME, cl-casp3 and β-actin.

1 lane: marker； 2 lane: MGC803 DMSO; 3 lane: MGC803 SIM; 4 lane: MGC803 SIM+DMPD; 5 lane: MGC803 SIM+DMLD
*: nonspecific bands.

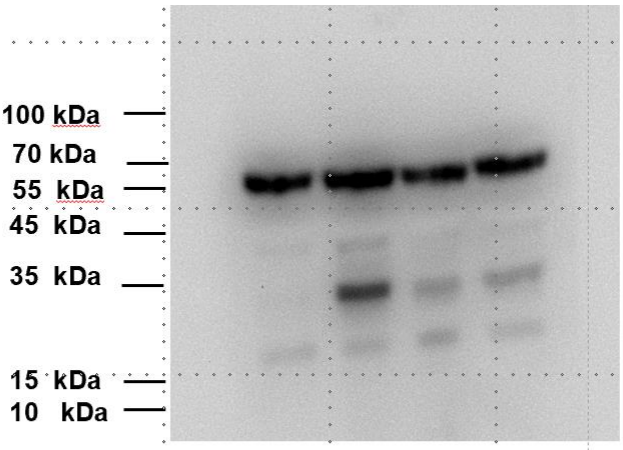

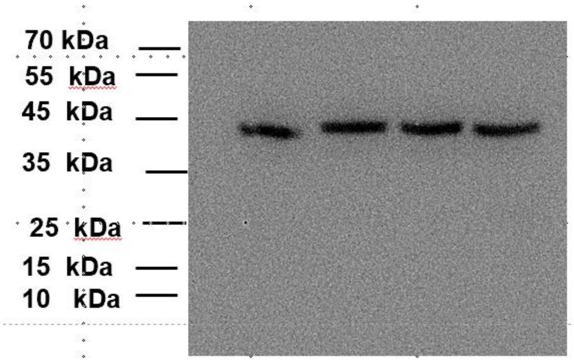

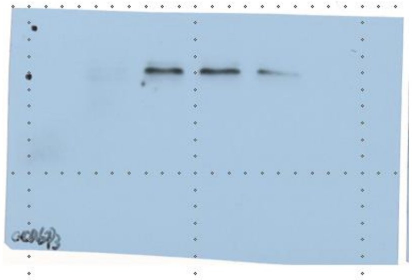

Figure 3

Figure 3B

The raw data of figure 3B was in Fig 3B.xlsx.

The protein expression of GSDME and β-actin.

1 lane: marker； 2 lane: MGC803 DOX (-); 3 lane MGC803 DOX (+)

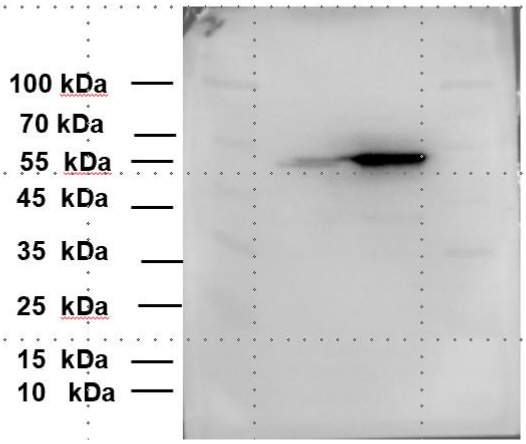

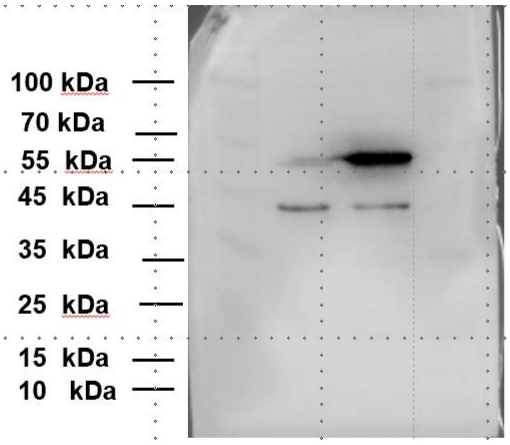

1 lane: marker；2 lane: HGC-27 DOX (-); 3 lane HGC-27 DOX (+)

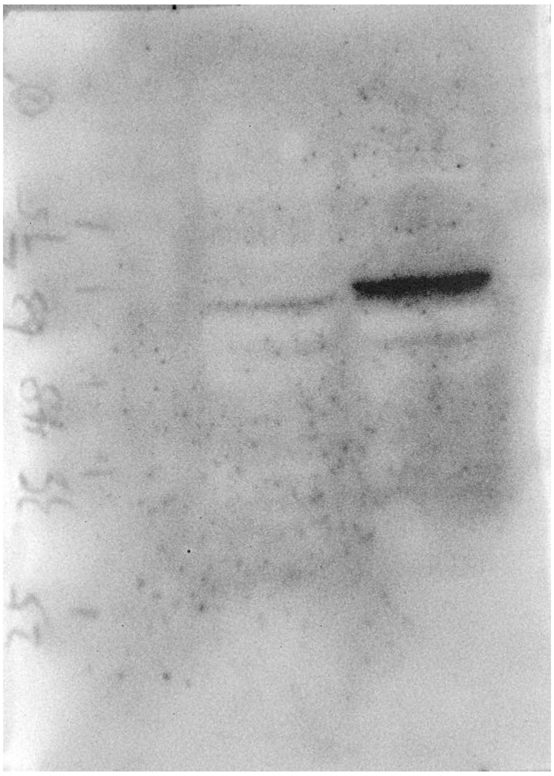

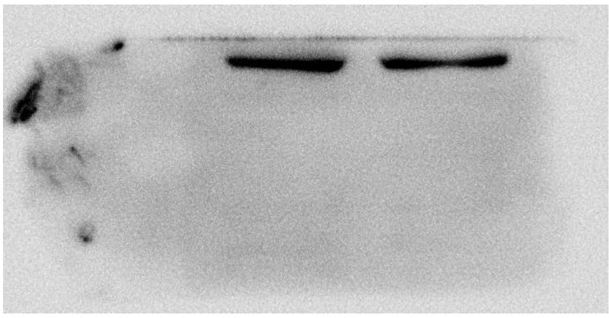

Figure 3C

First

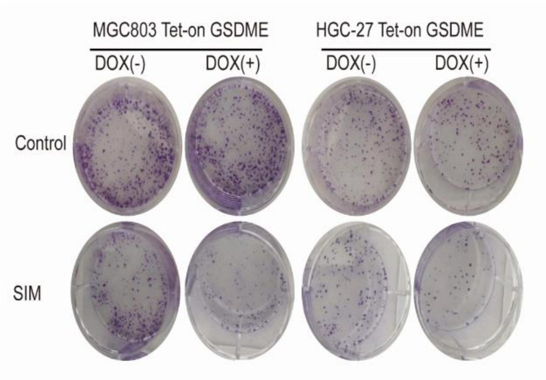

Second

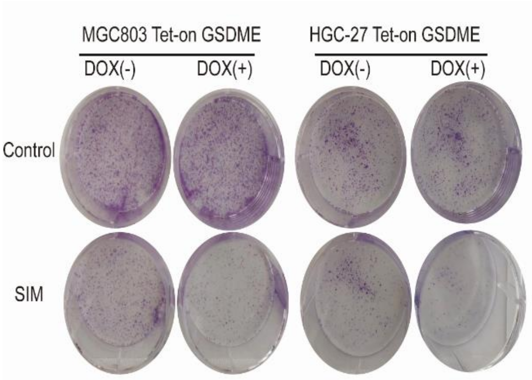

Third

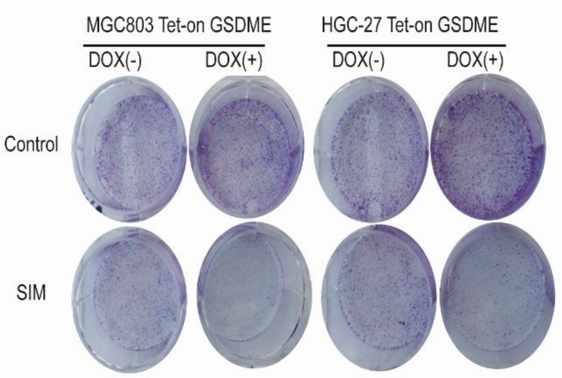

Figure 3G

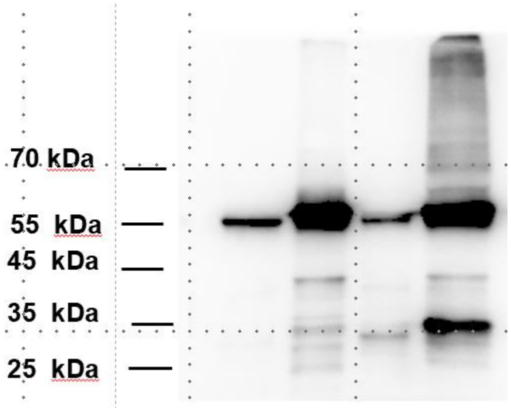
70 kDa
55 kDa
45 kDa
35 kDa
25 kDa

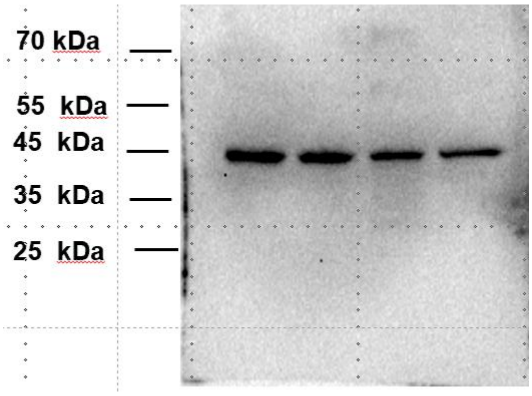
70 kDa
55 kDa
45 kDa
35 kDa
25 kDa

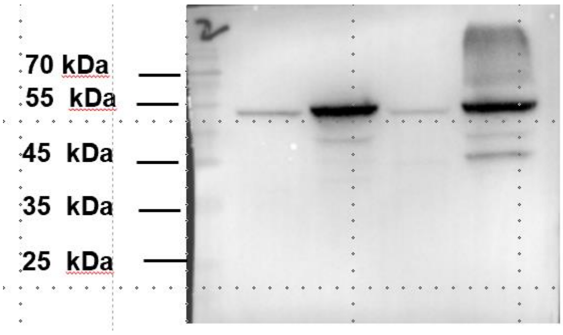
70 kDa
55 kDa
45 kDa
35 kDa
25 kDa

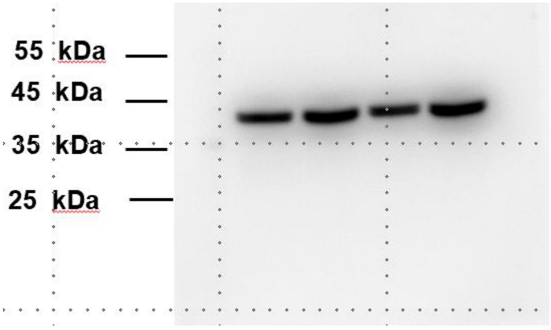
55 kDa
45 kDa
35 kDa
25 kDa

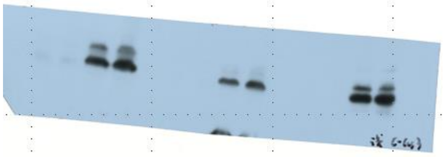

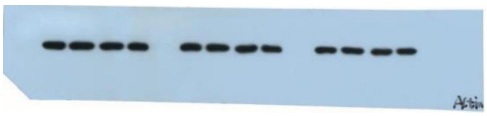

Figure 3H

MGC803 Tet-on GSDME

DOX(-)_Control_DIO | DOX(-)_Control_H | DOX(-)_Control_M

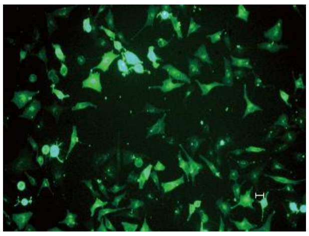
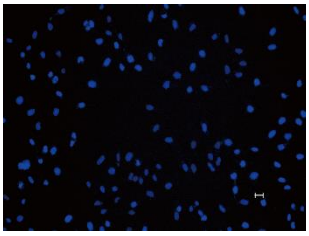
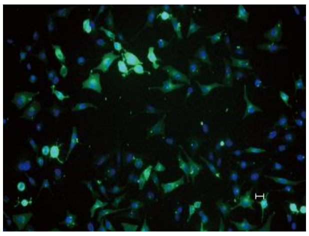

DOX(+)_Control_DIO | DOX(+)_Control_H | DOX(+)_Control_M

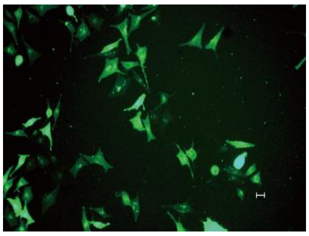
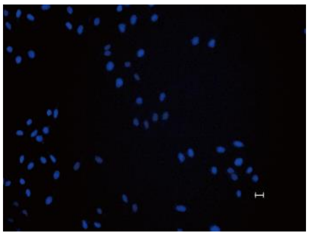
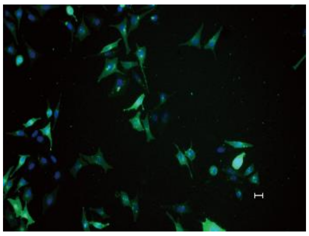

DOX(-)_SIM_DIO | DOX(-)_SIM_H | DOX(-)_SIM_M

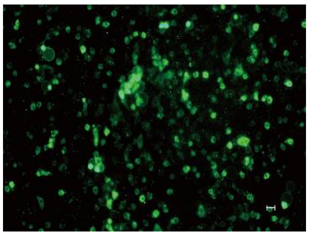
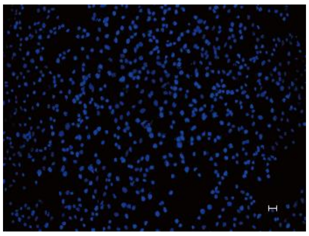
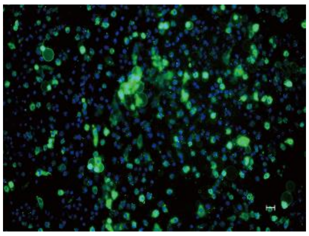

DOX(+)_SIM_DIO | DOX(+)_SIM_H | DOX(+)_SIM_M

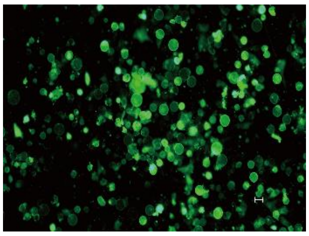
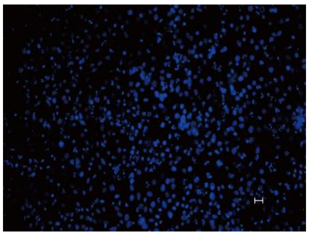
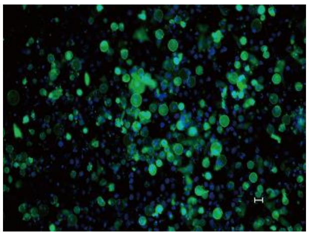

HGC-27 Tet-on GSDME

DOX(-)_Control_DIO | DOX(-)_Control_H | DOX(-)_Control_M

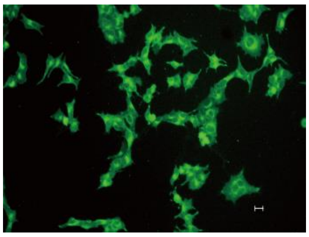
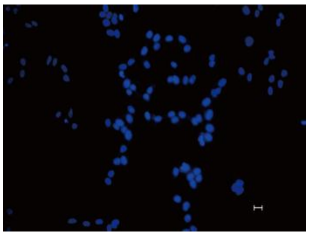
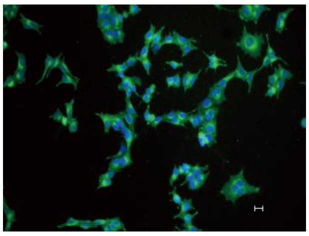

DOX(+)_Control_DIO | DOX(+)_Control_H | DOX(+)_Control_M

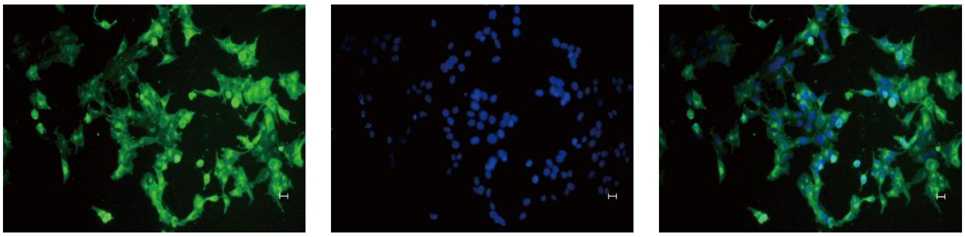

DOX(-)_SIM_DIO DOX(-)_SIM_H DOX(-)_SIM_M

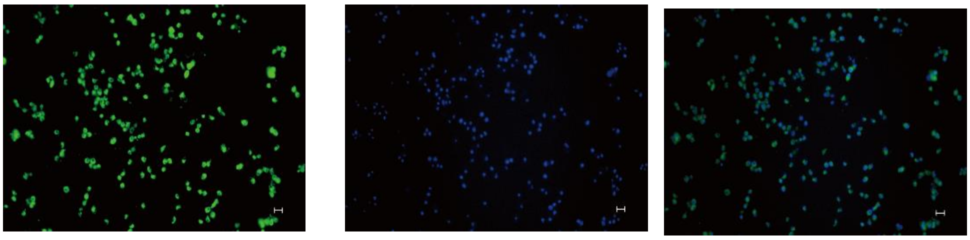

DOX(+)_SIM_DIO DOX(+)_SIM_H DOX(+)_SIM_M

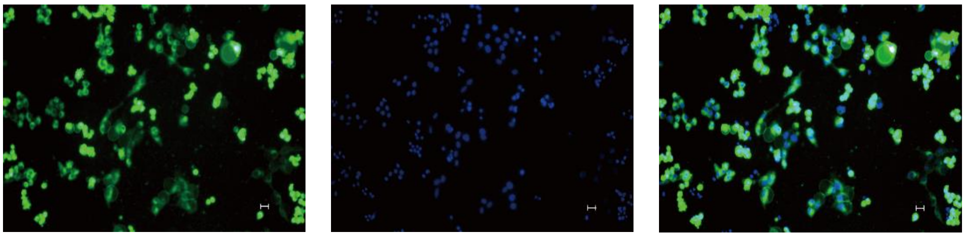

Figure 4

Figure 4B

DOX(-) 0d DOX(+) 0d DOX(-)_SIM 0d DOX(+)_SIM 0d

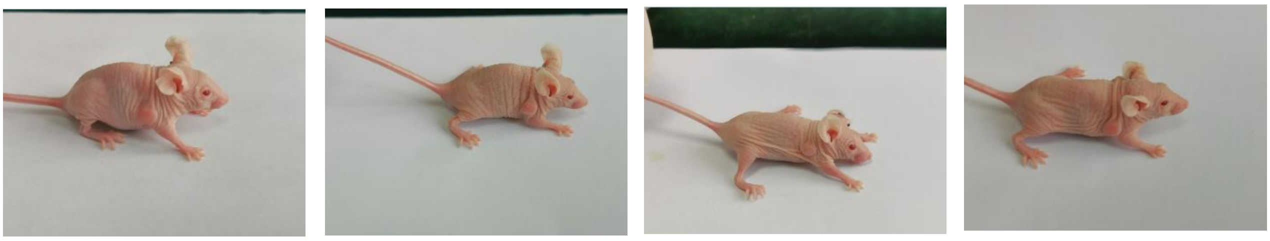

DOX(-) 7d DOX(+) 7d DOX(-)_SIM 7d DOX(+)_SIM 7d

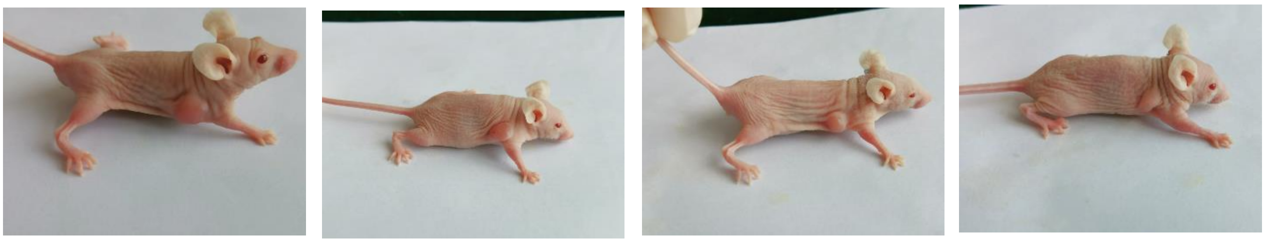

DOX(-)14d DOX(+) 14d DOX(-)_SIM 14d DOX(+)_SIM 14d

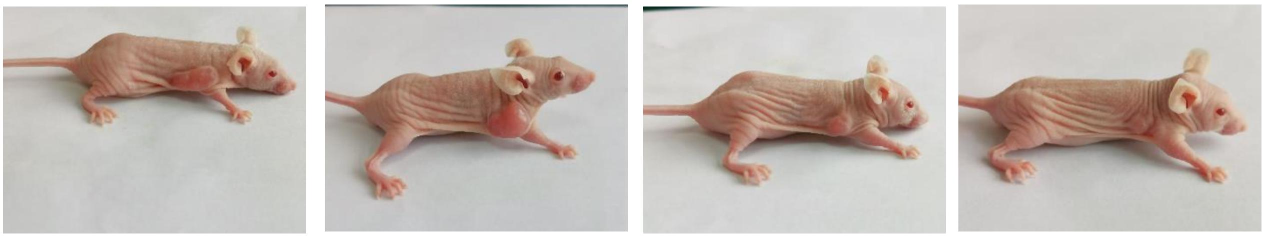

Figure 4C

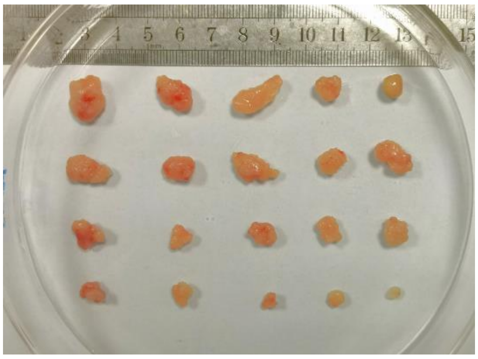

Figure 5

Figure 5A

H&E

DOX(-) DOX(+) DOX(-)_SIM DOX(+)_SIM

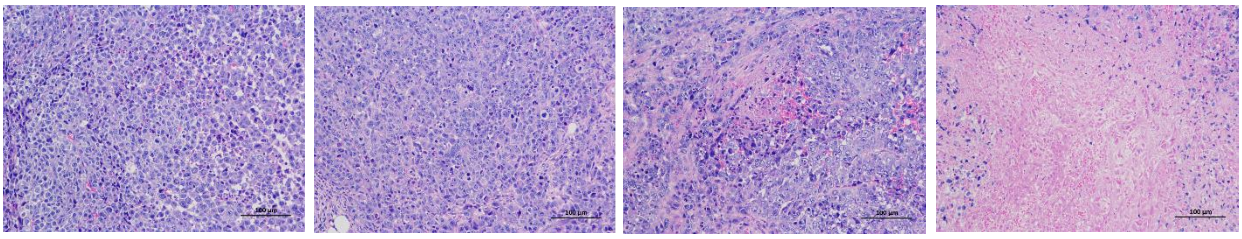

TUNEL staining

DOX(-)

TUNEL DAPI MERGE

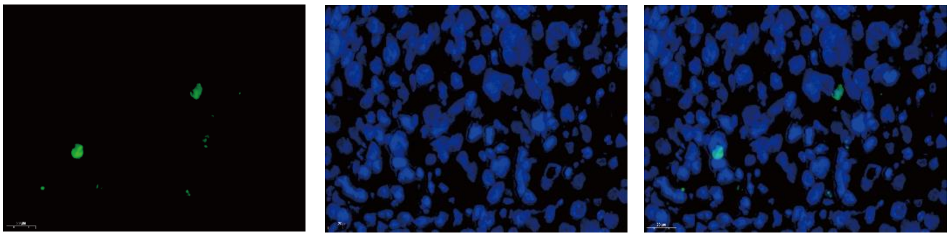

DOX(-)

TUNEL DAPI MERGE

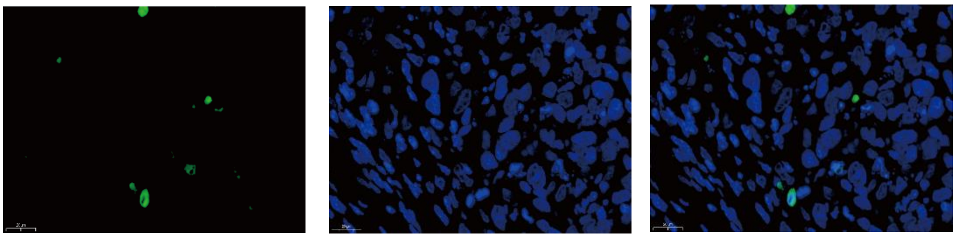

DOX(-)_SIM

TUNEL **DAPI** **MERGE**

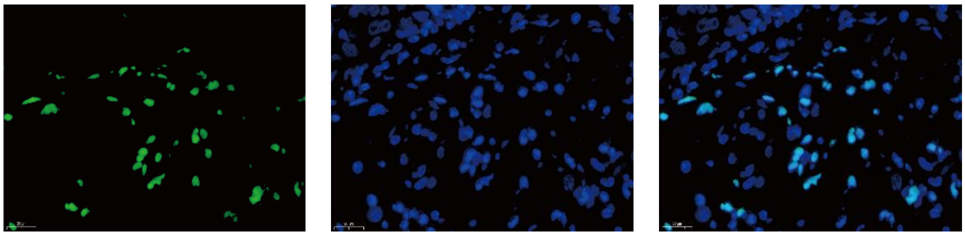

DOX(+)_SIM

TUNEL **DAPI** **MERGE**

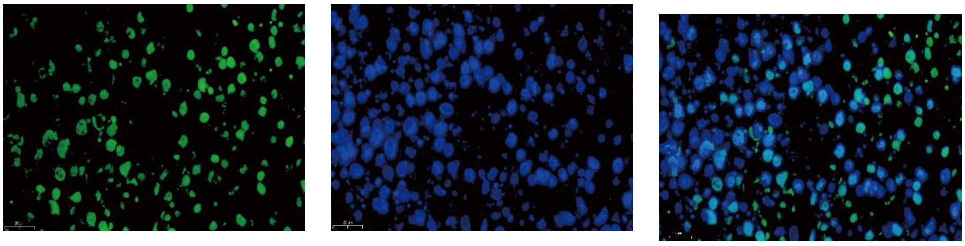

Figure 5B

The protein expression of GSDME, cl-casp 3 and β-actin.

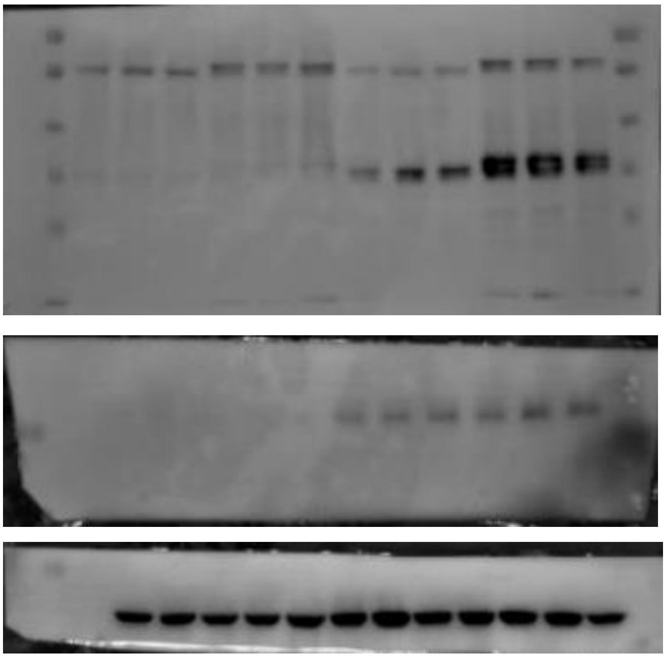

Figure 5C

Pictures are spliced directly without cropping in article.

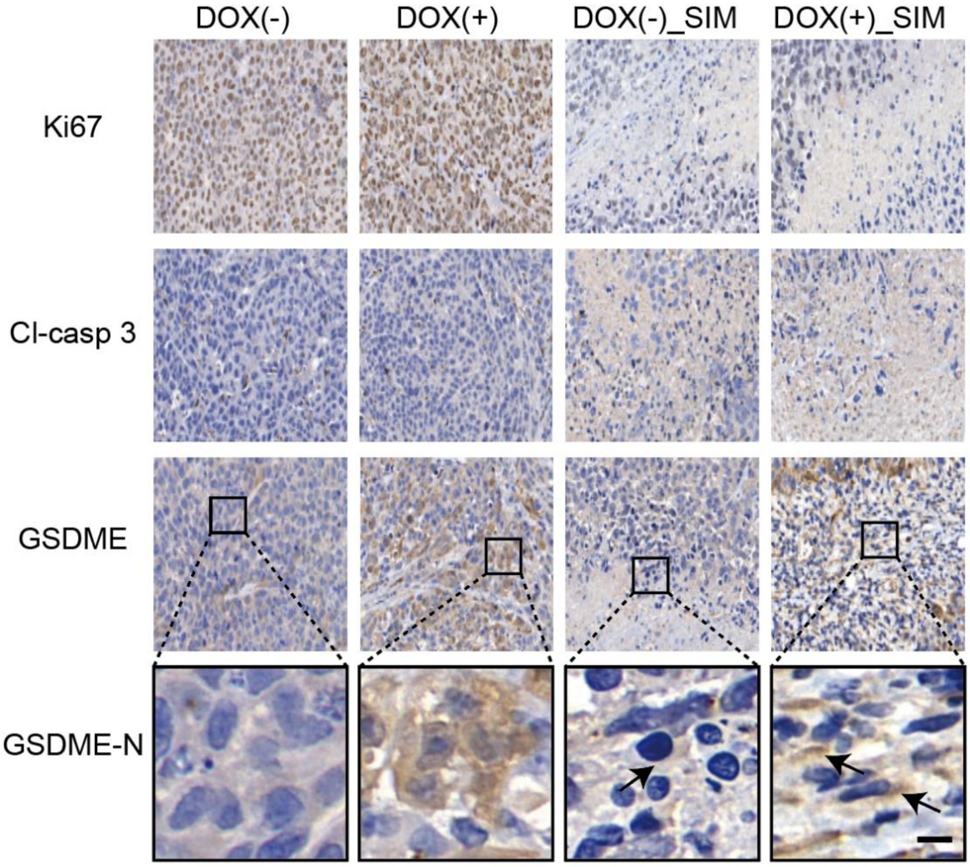

Figure 5D

Pictures are spliced directly without cropping in article.

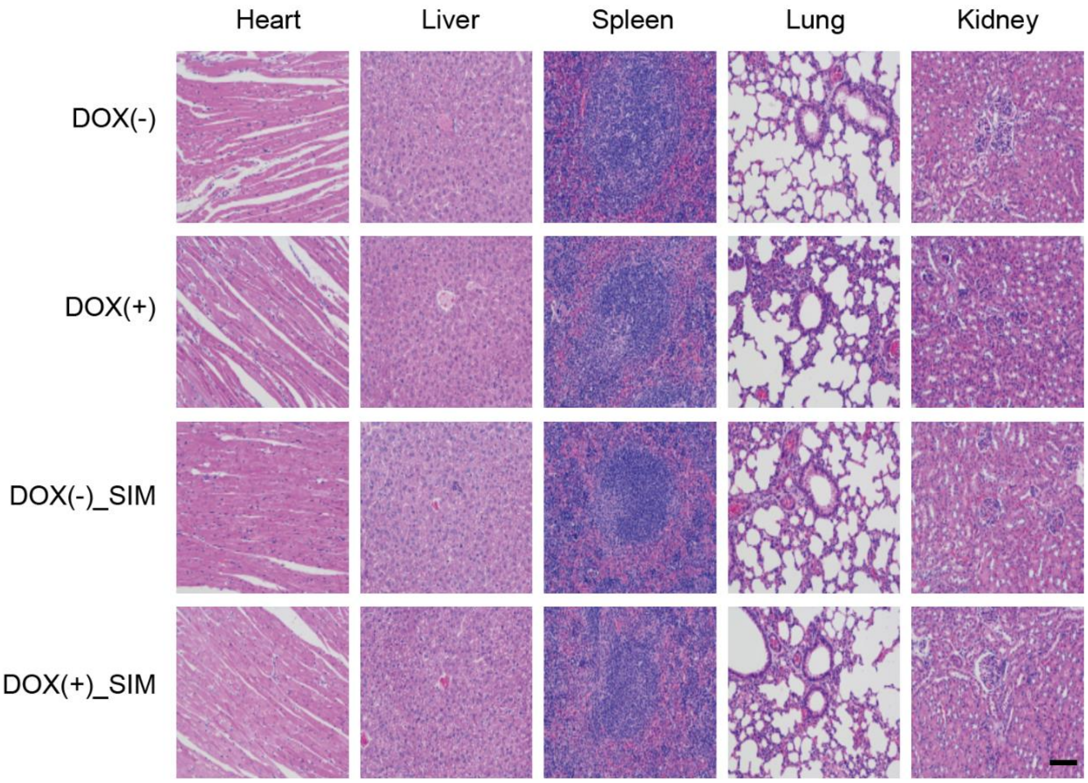

Figure 6

Figure 6A

The following figure is downloaded from the DiseaseMeth version 2.0 (http://biobigdata.hrbmu.edu.cn/diseasemeth/, accessed 30 August 2021).

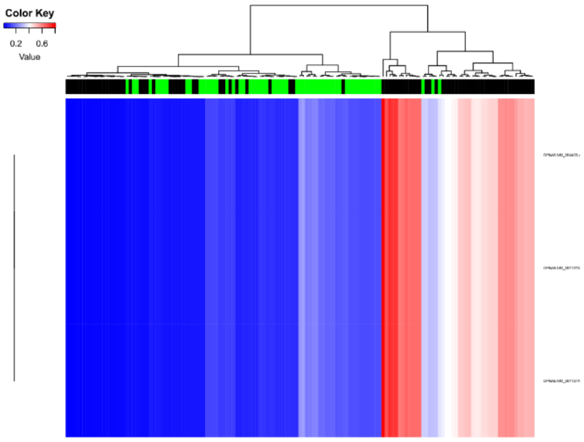

Figure 6B

H&E

N_4X **N_40X**

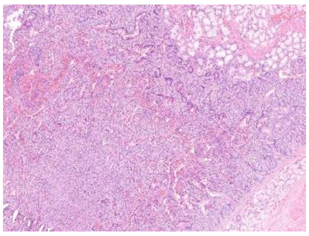
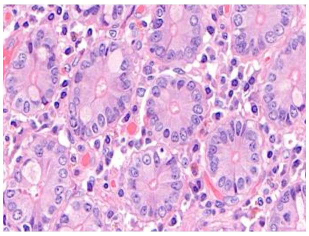

Tumor_4X **Tumor_40X**

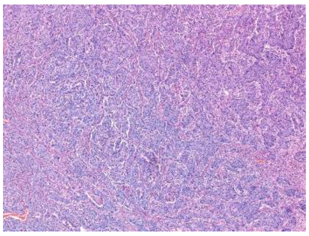
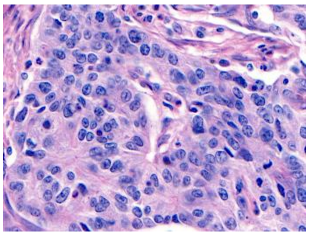

HIC GSDME

N_4X **N_40X**

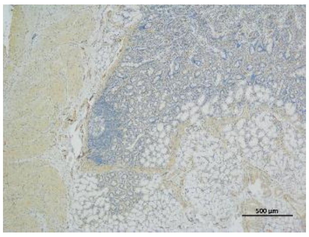
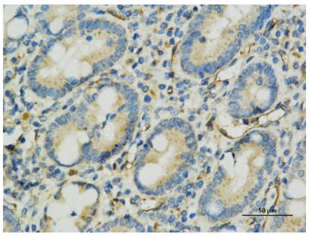

Tumor_4X **Tumor_40X**

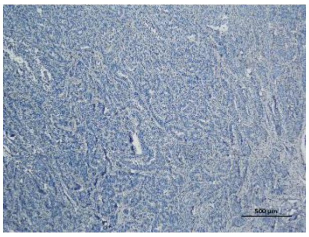
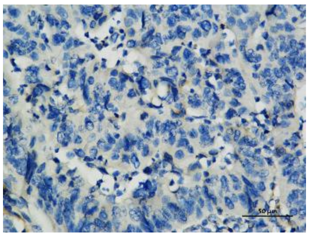

Figure 6D

The pictures of MSP.

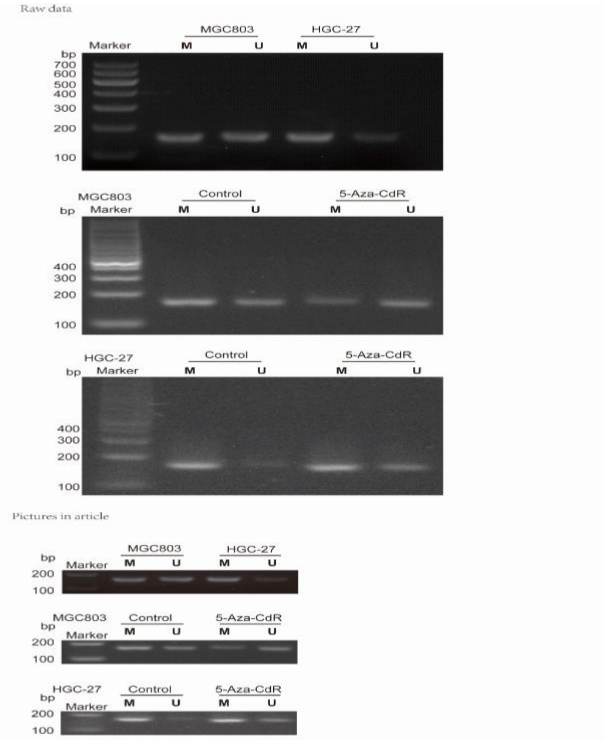

Figure 6I

MGC803

Control_DIO Control_H Control_M

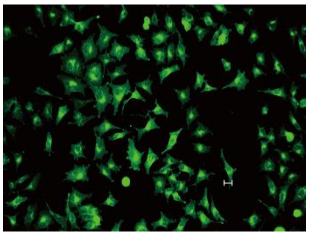
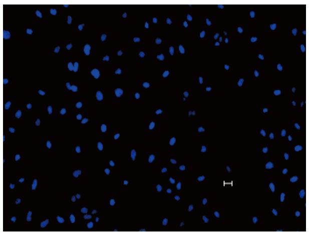
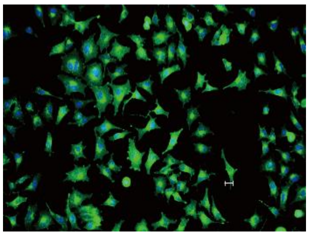

5-Aza_DIO 5-Aza_H 5-Aza_M

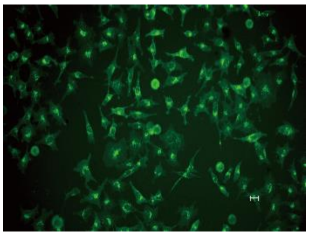
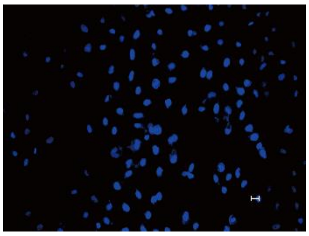
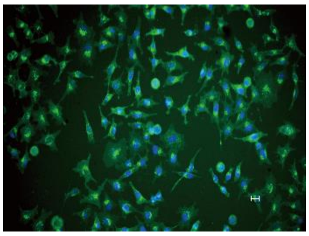

SIM_DIO SIM_H SIM_M

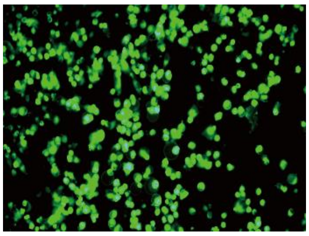
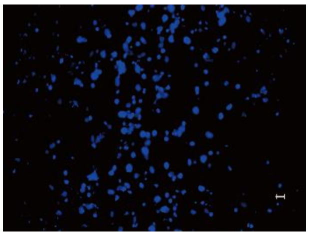
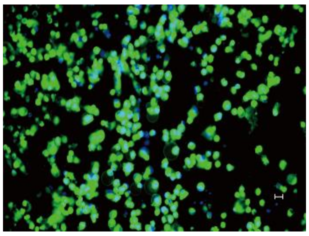

Combination_DIO Combination_H Combination_M

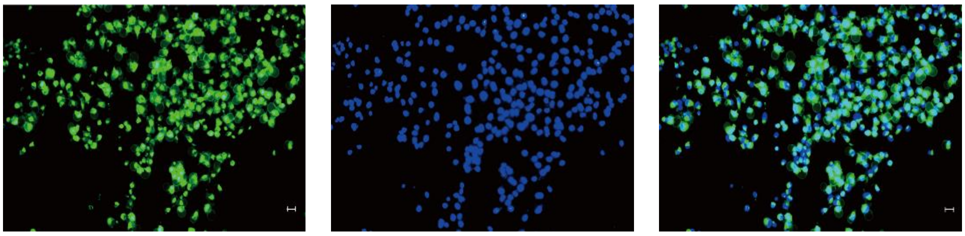

HGC-27

Control_DIO Control_H Control_M

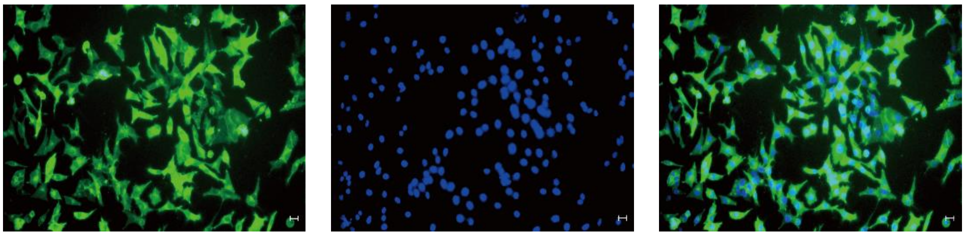

5-Aza_DIO 5-Aza_H 5-Aza_M

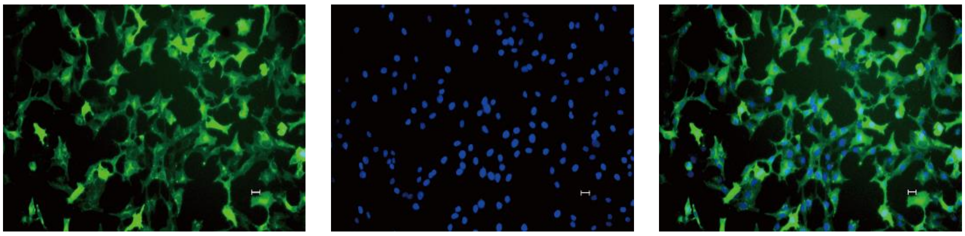

SIM_DIO SIM_H SIM_M

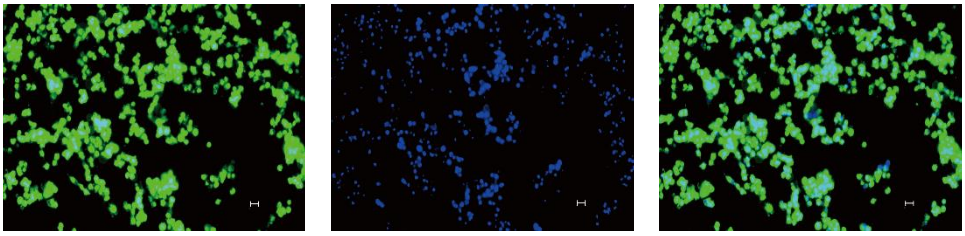

Combination_DIO Combination_H Combination_M

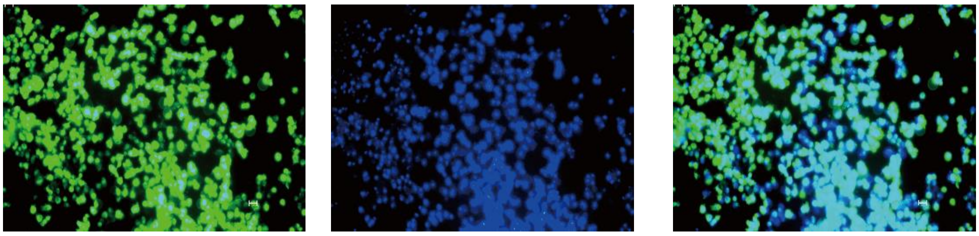

Figure 6J

Fig 6. J

The protein expression of GSDME, cl-casp 3 and β-actin.

1 lane: marker； 2 lane: MGC803 DMSO; 3 lane: MGC803 5-Aza; 4 lane: MGC803 SIM; 5 lane: MGC803 5-Aza+SIM

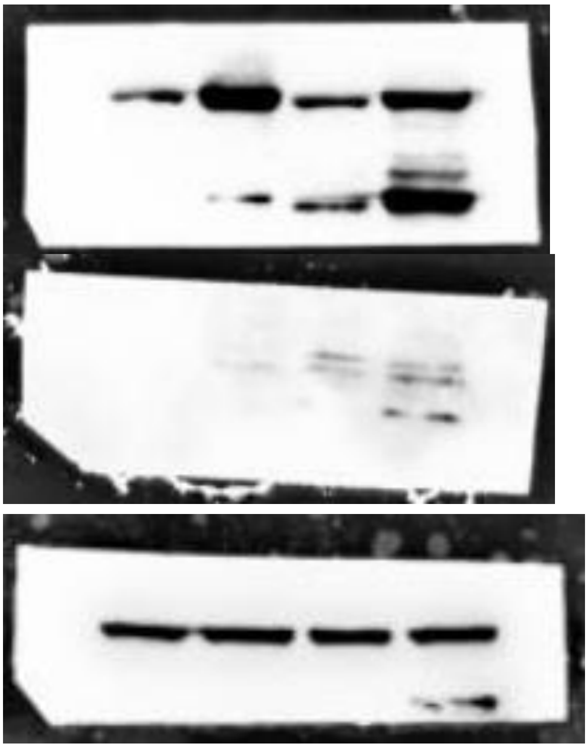

The protein expression of GSDME, cl-casp 3 and β-actin.

1 lane: marker；2 lane: HGC-27 DMSO; 3 lane: HGC-27 5-Aza; 4 lane: HGC-27 SIM; 5 lane: HGC-27 5-Aza+SIM

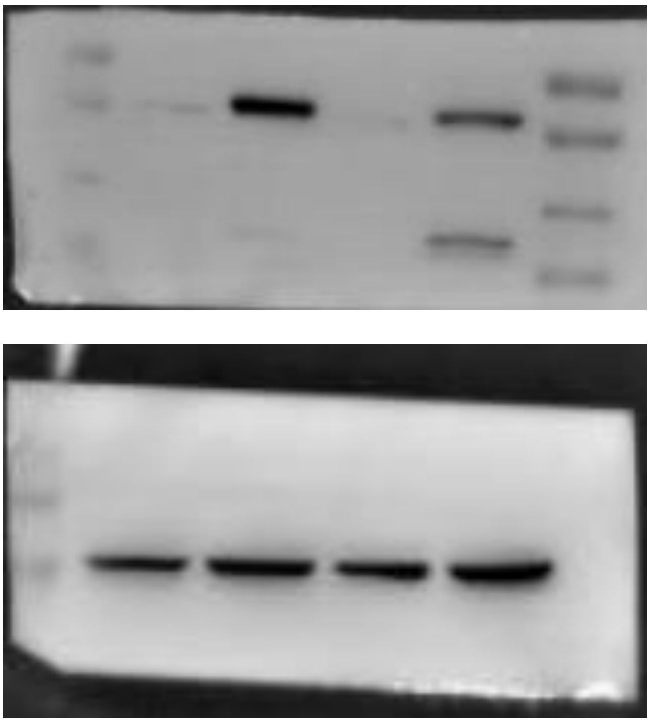

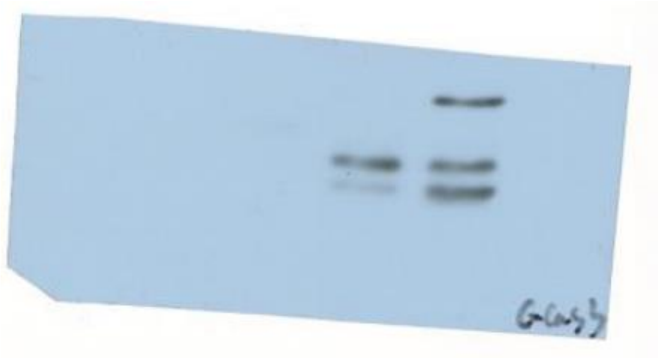

Figure 7

Figure 7B

Vehicle 0d

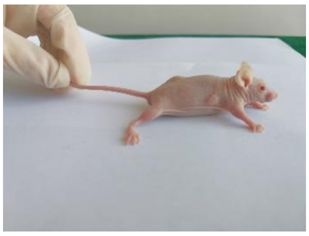

Vehicle 7d

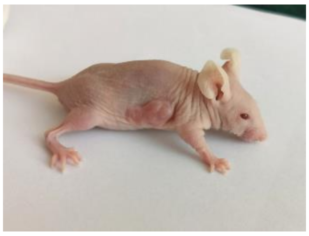

Vehicle 14d

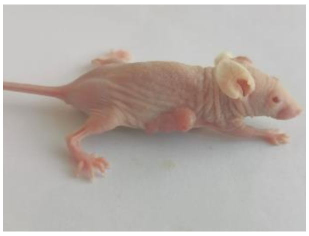

5-Aza 0d

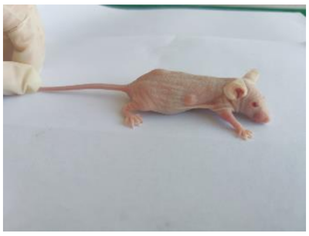

5-Aza 7d

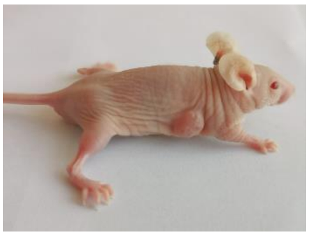

5-Aza 14d

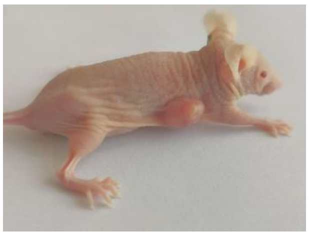

SIM 0d

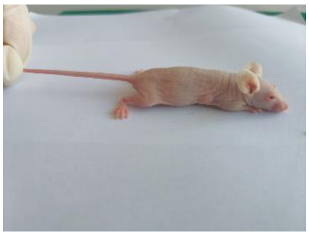

SIM 7d

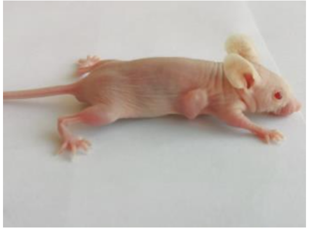

SIM 14d

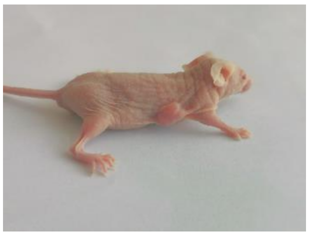

Combination 0d

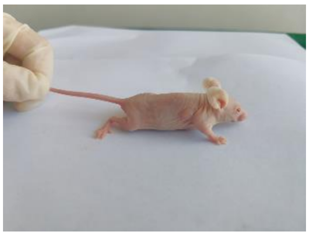

Combination 7d

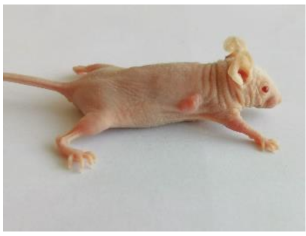

Combination 14d

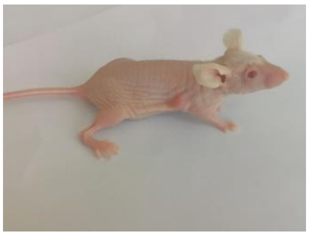

Figure 7C

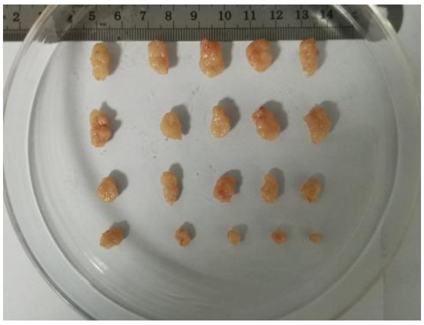

Figure 7G

The protein expression of GSDME, cl-casp 3 and β-actin.

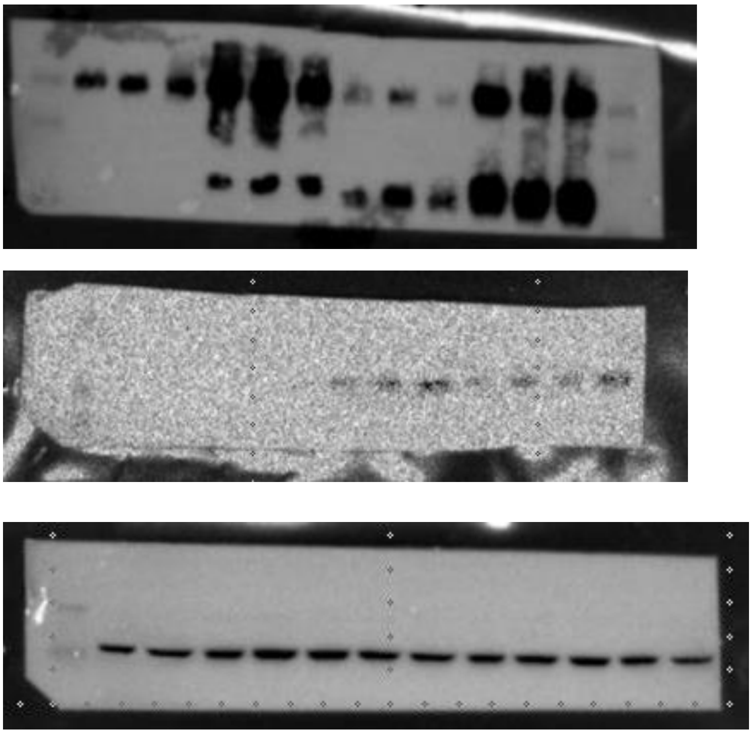

Figure 8

Figure 8A

H&E

Vehicle **5-Aza** **SIM** **Com**

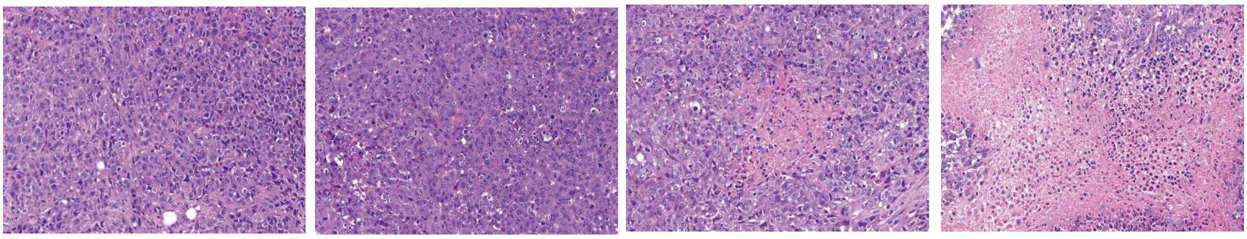

Vehicle_ki67 **5-Aza_ki67** **SIM_ki67** **Com_ki67**

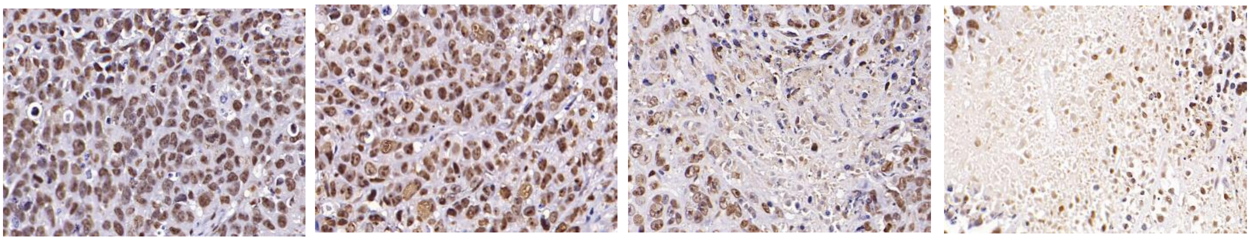

TUNEL staining

Vehicle_ TUNEL Vehicle_ DAPI Vehicle_ MERGE

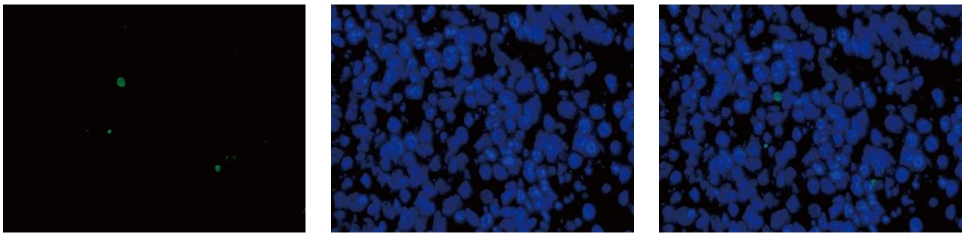

5-Aza_TUNEL 5-Aza_DAPI 5-Aza _MERGE

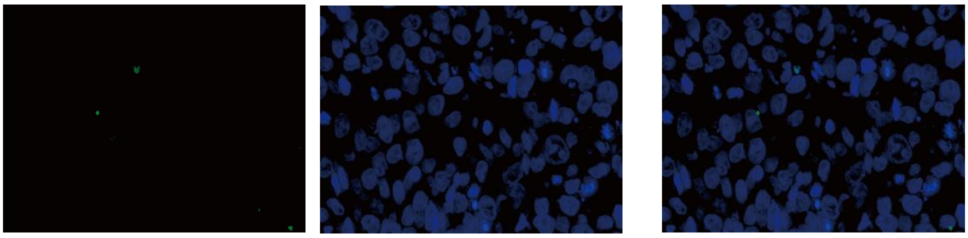

SIM_TUNEL SIM_DAPI SIM _MERGE

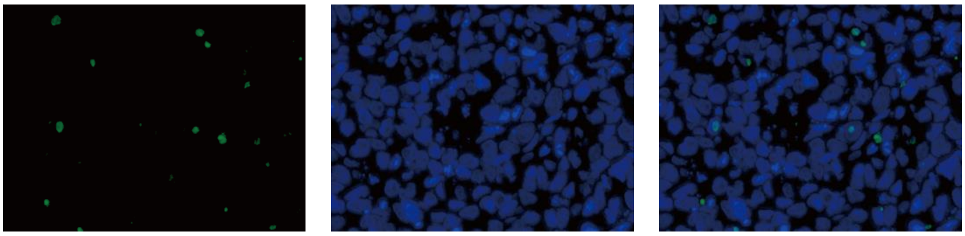

Com_ TUNEL Com_ DAPI Com _MERGE

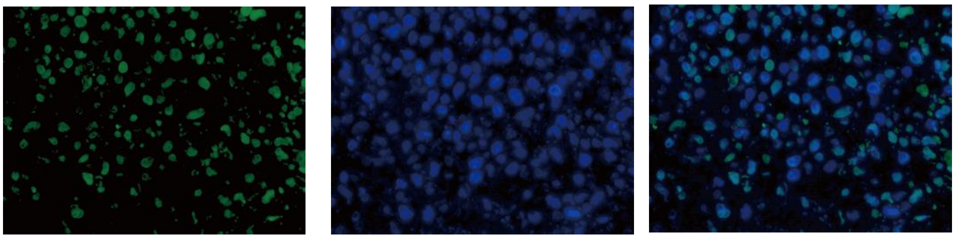

Figure 8B

Vehicle-heart Vehicle-liver Vehicle-spleen

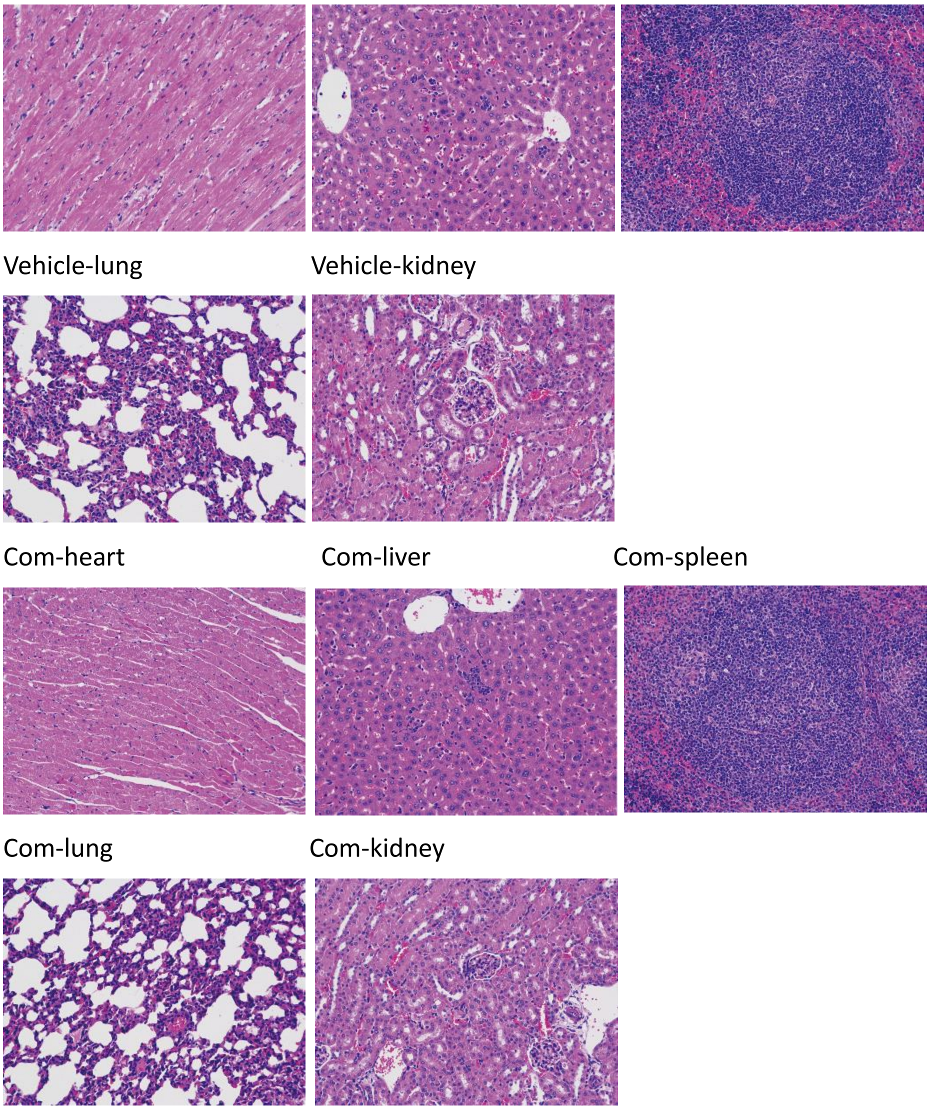
Vehicle-lung
Vehicle-kidney
Com-heart
Com-liver
Com-spleen
Com-lung
Com-kidney
