# Supplementary material for: Antitumor Effect of Simvastatin in Combination With DNA Methyltransferase Inhibitor on Gastric Cancer via GSDME-Mediated Pyroptosis
Source: Front Pharmacol. 2022 Apr 20;13:860546. doi: 10.3389/fphar.2022.860546 (PMC9065610; doi:10.3389/fphar.2022.860546)
Supplement: Supplementary file 2 [file DataSheet1.PDF]

Supplementary Figures

Supplementary Figure 1

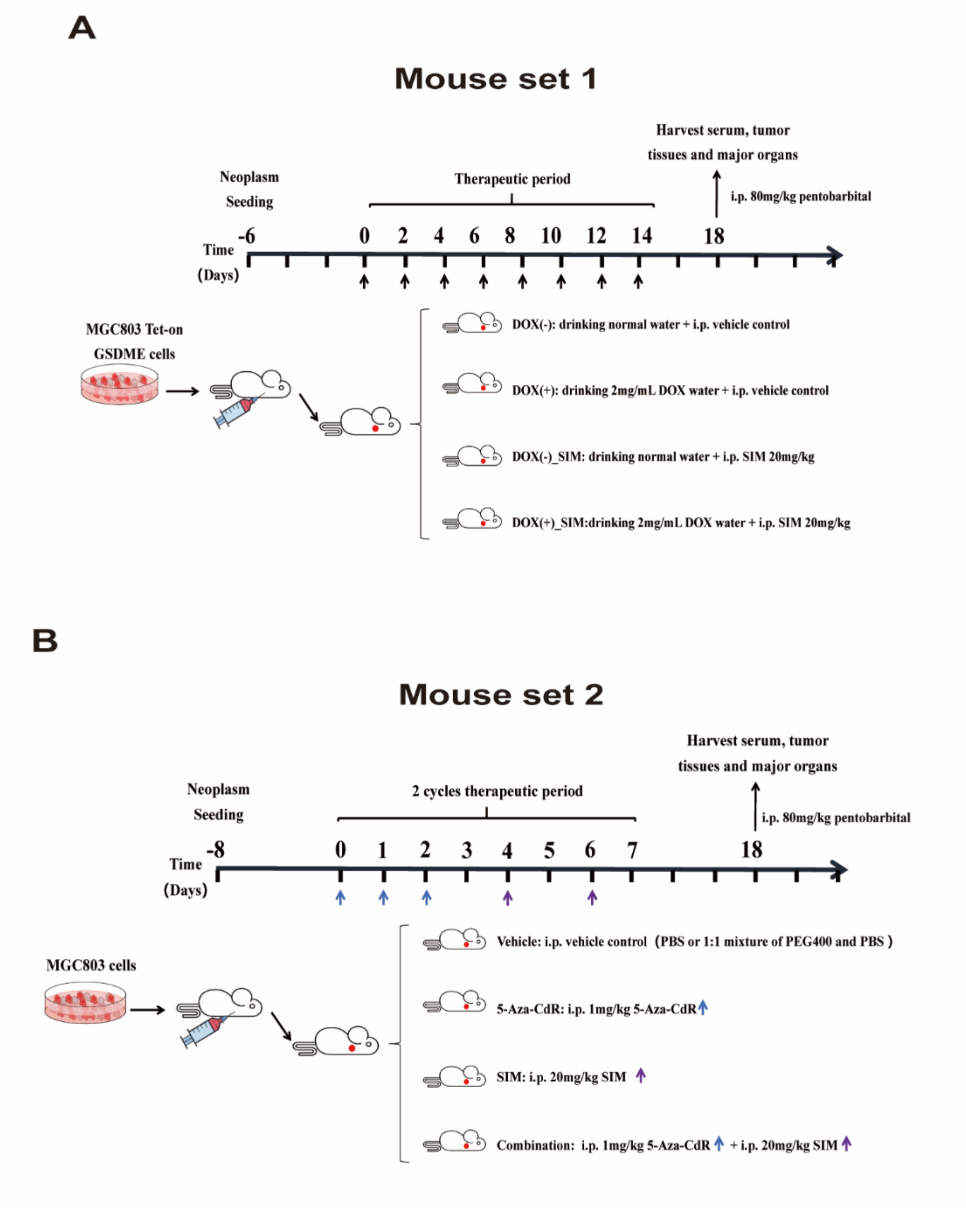

Supplementary Figure 1 Schematic of treatment regimens. (A) Female BALB/c mice 5 weeks of age implanted subcutaneously in the right flank with MGC803 Tet-on GSDME cells (2×10^6 cells/100 µl PBS; mouse set 1). (B) Female BALB/c mice 5 weeks of age implanted subcutaneously in the right flank with MGC803 cells (2×10^6 cells/100 µl PBS, mouse set 2). When the tumors reached a volume of approximately 60 mm^3, the mice were randomly divided into four groups (n=5). Groups in set 1 were DOX (-) vehicle control, DOX (+) GSDME overexpression, DOX (-) with SIM, and DOX (+) with SIM. Groups in set 2 were vehicle control, 5-Aza-CdR, SIM and SIM+5-Aza-CdR group. Mice in each group were treated according to the assigned treatment schematic (illustrated above).

Supplementary Figure 2

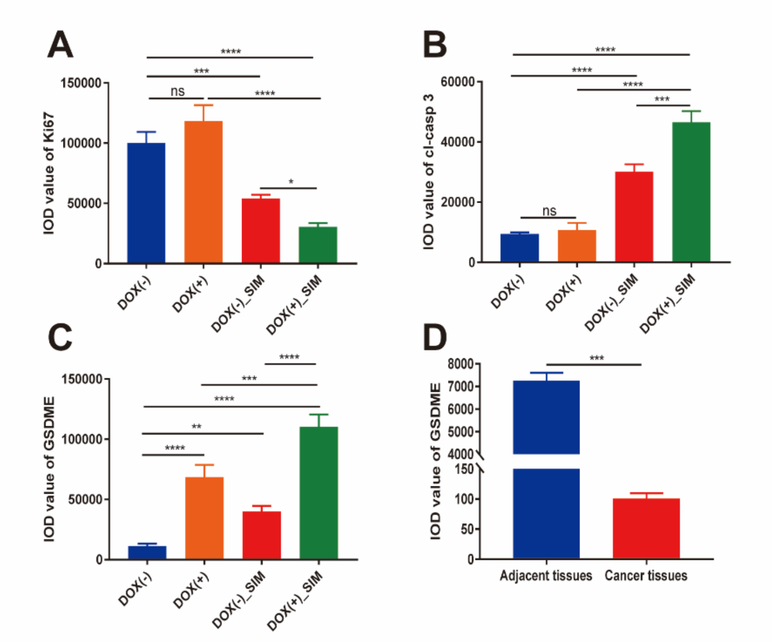

[1]

Supplementary Figure 2 Bar graphs show the quantification results of immunohistochemistry (IHC). The quantification results of immunohistochemistry for Ki67 (A), Cl-casp 3 (B), GSDME (C), in tissues of nude mice. (D) The quantification results of immunohistochemistry for GSDME protein in adjacent noncancerous gastric tissues and GC tissues. IOD, integrated optical density (n = 3). A *p*-value < 0.05 was considered significant (** $p < .01$, *** $p < .001$ and **** $p < .0001$), and $p > .05$ not significant (ns).

Supplementary Figure 3

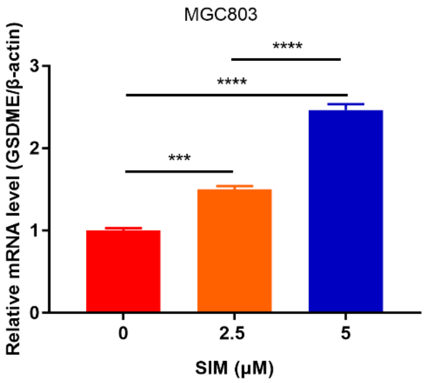

Supplementary Figure 3 MGC803 cells were treated with different doses of SIM for 48h, the expression of GSDME gene was detected by RT-qPCR and expressed as mean ± SD (n = 3). DMSO served as a control group. A p-value < 0.05 was considered significant (*** $p < .001$ and **** $p < .0001$).

Supplementary Tables

Supplementary Table 1 The correlation between clinicopathological parameters and the expression level of GSDME in 20 gastric cancer patients

**Variables**	**n**	**the expression level of GSDME[a]**		***p*-value**
		**High**	**Low**	
**Total**	20	10	10	
**Gender**				0.370
Male	11	7	4	
Female	9	3	6	
**Age**				0.656
≤60	10	6	4	
>60	10	4	6	
**Tumor size**				1.000
≤5cm	10	5	5	
>5cm	10	5	5	
**Differentiation**				0.628
Well/Moderate	6	4	2	
Poor	14	6	8	
**Tumor status**				0.650
T1-T2	12	7	5	
T3-T4	8	3	5	
**Lymph node invasion**				1.000
N0	3	1	2	
N1-N3	17	9	8	
**Distant metastasis**				
M0	18	9	9	1.000
M1	2	1	1	

[a]The median ratio of the expression level of GSDME

Supplementary Table 2 All RT-qPCR primers (5' – 3') in this manuscript

Gene Name	Forward Primer	Reverse Primer
**GSDMD (homo)**	GCTGGTTATTGACTCTGACTTGGAC	GACCCCATCTGTCAGGAAGTT
**GSDME (homo)**	CCAGTTTTTATCCCTCACCCTTG	CAAACTTGCCCTCGTATTTCACA
**Caspase 3 (homo)**	TGGAAGCGAATCAATGGACTCT	TGAATGTTTCCCTGAGGTTTGC
**β-actin**	CACCCAGCACAATGAAGATCAAGAT	CCAGTTTTTAAATCCTGAGTCAAGC

Supplementary Table 3 The information of primary antibody

Antibodies	Company	Catalog Number	Host	Reactivity	Working Concentration
**GSDMD**	Proteintech	20770-1-AP	Rabbit	Human, Rat	1：2500 (WB)
**DFNA5/GSDME - N-terminal**	Proteintech	13075-1-AP	Rabbit	Mouse, Human	1：250 (IHC)
**DFNA5/GSDME - N-terminal**	Abcam	ab215191	Rabbit	Mouse, Rat, Human	1：1000 (WB)
**Caspase 3**	CST	#14220	Rabbit	Human, Rat, Mouse, Monkey	1：1000 (WB)
**cl-caspase 3**	CST	#9664	Rabbit	Human	1：1000 (WB)<br>1:200 (IHC)
**Ki67**	Servicebio	GB111499	Rabbit	Mouse, Rat, Human	1：250 (IHC)
**β-actin**	Proteintech	66009-1-lg	Mouse	Human, Rat, Mouse, Monkey	1：10000 (WB)

Supplementary Table 4 Primer pairs specific to methylated (M) and unmethylated (U) GSDME promoter sequences (5' – 3')

Gene Name	Forward Primer	Reverse Primer
**GSDME-M**	AGGAGGCGTCGTTTTTAAATTTTA	ATTACTAACGACCCAACGCTCAA
**GSDME-U**	GAGGAGGTGTTGTTTTTAAATTTTA	AATTACTAACAACCCAACACTCAAC

Supplementary Table 5 Clinical chemistry parameters for combination therapy of simvastatin and overexpression-GSDME in vivo

Groups	ALT(U/L)	AST(U/L)	CRE(μmol/L)	BUN（mg/dL）
**DOX (-)**	53.799±20.188	183.127±50.641	9.516±0.805	22.475±1.694
**DOX (+)**	47.261±4.449	144.624±7.728	7.566±0.450	23.025±1.379
**DOX (-) _SIM**	46.594±11.509	174.222±49.573	9.416±1.960	21.523±3.301
**DOX (+) _SIM**	46.682±0.764	135.180±30.624	9.836±2.388	23.427±3.929

ALT alanine aminotransferase, AST aspartate aminotransferase, CRE creatinine,BUN blood urea nitrogen

Supplementary Table 6 Clinical chemistry parameters for combination therapy of simvastatin and 5-Aza-CdR in vivo

Groups	ALT(U/L)	AST(U/L)	CRE(μmol/)	BUN（mg/dL）
**Vehicle**	51.215±2.205	156.824±11.531	9.920±1.988	25.229±0.471
**Combination**	55.729±3.933	195.467±73.001	8.571±1.061	27.534±3.878
